# Two-step anti-cooperative self-assembly process into defined π-stacked dye oligomers: insights into aggregation-induced enhanced emission†

**DOI:** 10.1039/d1sc03813c

**Published:** 2021-08-17

**Authors:** Yvonne Vonhausen, Andreas Lohr, Matthias Stolte, Frank Würthner

**Affiliations:** Institut für Organische Chemie, Universität Würzburg Am Hubland 97074 Würzburg Germany wuerthner@uni-wuerzburg.de; Center for Nanosystems Chemistry (CNC), Bavarian Polymer Institute (BPI), Universität Würzburg Theodor-Boveri-Weg 97074 Würzburg Germany

## Abstract

Aggregation-induced emission enhancement (AIEE) phenomena received great popularity during the last decade but in most cases insights into the packing structure – fluorescence properties remained scarce. Here, an almost non-fluorescent merocyanine dye was equipped with large solubilizing substituents, which allowed the investigation of it's aggregation behaviour in unpolar solvents over a large concentration range (10^−2^ to 10^−7^ M). In depth analysis of the self-assembly process by concentration-dependent UV/Vis spectroscopy at different temperatures revealed a two-step anti-cooperative aggregation mechanism. In the first step a co-facially stacked dimer is formed driven by dipole–dipole interactions. In a second step these dimers self-assemble to give an oligomer stack consisting of about ten dyes. Concentration- and temperature-dependent UV/Vis spectroscopy provided insight into the thermodynamic parameters and allowed to identify conditions where either the monomer, the dimer or the decamer prevails. The centrosymmetric dimer structure could be proven by 2D NMR spectroscopy. For the larger decamer atomic force microscopy (AFM), diffusion ordered spectroscopy (DOSY) and vapour pressure osmometric (VPO) measurements consistently indicated that it is of small and defined size. Fluorescence, circular dichroism (CD) and circularly polarized luminescence (CPL) spectroscopy provided insights into the photofunctional properties of the dye aggregates. Starting from an essentially non-fluorescent monomer (*Φ*_Fl_ = 0.23%) a strong AIEE effect with excimer-type fluorescence (large Stokes shift, increased fluorescence lifetime) is observed upon formation of the dimer (*Φ*_Fl_ = 2.3%) and decamer (*Φ*_Fl_ = 4.5%) stack. This increase in fluorescence is accompanied for both aggregates by an aggregation-induced CPL enhancement with a strong increase of the *g*_lum_ from ∼0.001 for the dimer up to ∼0.011 for the higher aggregate. Analysis of the radiative and non-radiative decay rates corroborates the interpretation that the AIEE effect originates from a pronounced decrease of the non-radiative rate due to π–π-stacking induced rigidification that outmatches the effect of the reduced radiative rate that originates from the H-type exciton coupling in the co-facially stacked dyes.

## Abstract 2

The self-assembly of a dipolar merocyanine into preferred dimers and small-sized chiral aggregates leads to enhanced emission due to a reduced non-radiative rate as well as amplified circular polarized luminescence.

## Graphical abstract

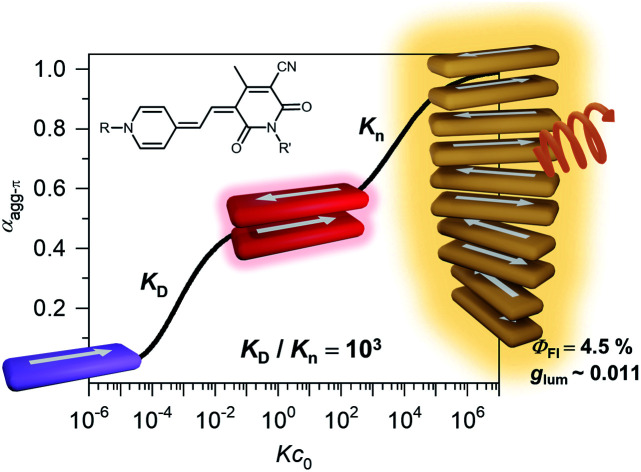

## Introduction

**Fig. 1:**
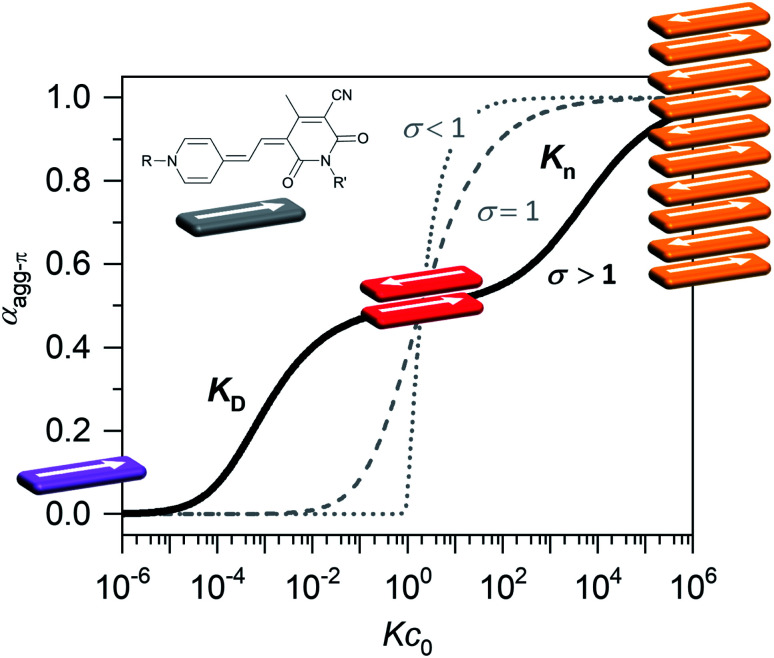
Degree of aggregated π-faces (*α*_agg-π_) as a function of *Kc*_0_ according to the isodesmic (dashed grey line), cooperative (dotted grey line) and anti-cooperative (solid black line) model with a dimer nucleus, governed by the cooperativity parameter *σ* = *K*_D_/*K*_n_. Inset shows the dipolar chromophore of merocyanine **1**.

Organic materials, especially dyes and pigments, have gained considerable interest for high performance technology applications like organic solar cells or other optoelectronic devices^1^ as they promise low costs and easy and versatile processing. The desired properties of those organic functional materials are, however, tightly connected to interactions between the molecules in the solid state. Therefore, predicting and controlling the intermolecular arrangement along with understanding structure–property relationships, is a key step toward tailor-made functional organic materials.^2,3^ For this, defined dye aggregates in solution can play an important role as model systems of reduced complexity. Already by investigating rather simple dimer systems, fundamental insights can be gained on processes like aggregation-induced enhanced emission (AIEE),^4^ symmetry-breaking charge separation^5^ or singlet fission.^6^ For a better understanding, especially of solid-state materials, however, the investigation of larger systems is crucial. Oligomeric systems of defined size and geometry have the advantage, that multi-chromophore interactions can be studied in solution without the drawbacks of possible phase separation or gel formation that can occur upon formation of polymeric structures. The deliberate formation of defined-sized aggregate stacks, however, is quite challenging. Good results were obtained by connecting dye molecules to covalent backbones^7–9^ that favour a specific arrangement or by using templating effects.^10,11^ Another, less explored, method to form small-sized aggregates, which does not require the addition of templates or the covalent linking of chromophores, is taking advantage of an anti-cooperative aggregation mechanism. The self-assembly mechanism,^12^ which is encoded in the molecular building blocks, dictates important properties like stability, size and size distribution of the emerging aggregates.^13,14^ Three classical cases are differentiated: isodesmic, cooperative and anti-cooperative aggregation (Fig. 1).

In the case of isodesmic aggregation, all binding sites (*e.g.* both π-faces of a chromophore) are equivalent and independent and thus the binding constant *K* is equal for every individual binding event upon self-assembly. This usually leads to oligomeric aggregates with large polydispersity. In case of cooperative aggregation, first an unfavourable nucleation step (*e.g.* dimer formation, *K*_D_) has to occur at a critical concentration or temperature before polymerization (*K*_n_) takes place starting from a relatively small amount of nuclei (*K*_D_ < *K*_n_). The resulting aggregates are much longer, *i.e.* supramolecular polymers, compared to those obtained by isodesmic aggregation.^15^ Because these features are often desirable for functional supramolecular polymers cooperative self-assembly has been widely explored.^16,17^ To study intermolecular interactions and their influence on optical or electronic properties, however, small and well-defined aggregates of a discrete number of chromophores are preferred. Here an anti-cooperative aggregation mechanism can be advantageous, since it is characterized by a preferred nucleus formation and less favoured further aggregation (*K*_D_ > *K*_n_) of those nuclei. Thus, rather small-sized aggregates with a narrow size distribution can be obtained.^18–20^ When plotting the degree of aggregated π-faces (*α*_agg-π_) as a function of the dimensionless concentration *Kc*_0_, a characteristic curve is obtained for the anti-cooperative model (Fig. 1, black line), with a plateau at the concentration range, where predominantly the nucleus species is present (*α*_agg-π_ = 0.5 for dimer case). Anti-cooperativity requires special molecular features that allow two competing intermolecular interactions, a strong one (*e.g.*, H-bonding^21,22^ or dipole–dipole^23^ interactions) leading to nucleus formation and a weaker one causing further self-assembly (often dispersion forces). Alternatively, the reduced binding strength for further assembly of the nuclei is caused by increasing sterical hindrance of bulky substituents^19,24–28^ or electrostatic repulsion.^18^ In this case smaller stack sizes of less than twenty chromophores have been realized. Clearly, such step-wise aggregation also provides interesting possibilities for the construction of complex structures out of relatively simple molecular building blocks,^21^ which can bring us one step closer toward the engineering of sophisticated supramolecular nanostructures as they can currently only be found in nature.^29^

Here we will show that the utilization of both concepts, *i.e.* preferential nucleation and sterical encumbrance to limit growth, can afford a hierarchical growth process to realize upon increasing concentration two defined aggregate species whose functional properties could be characterized. A most suitable class of molecules for the exploration of this concept are dipolar merocyanines^30^ which provide a textbook example for anti-cooperative self-assembly. Because of their significant zwitterionic character and the resulting large molecular ground state dipole moment (*μ*_g_) of ∼17 D for the dipolar chromophore of merocyanine **1**, dimerization is favoured due to strong, directional dipole–dipole interactions between antiparallel oriented chromophores.^31^ Since the molecular ground state dipole moments compensate each other in the dimer, further aggregation is only driven by the weaker dispersion forces between the remaining two free π-faces. Owing to the significant decrease of binding strength stacking of more than two chromophores has never been observed for individual merocyanines in solution but only for bis-merocyanines with tweezer like structures^32–34^ or foldamers^35^ whose self-assembly could be directed into a broad variety of supramolecular oligomers and polymers. Here, by introduction of a large solubilizing substituent (“wedge”)^36^ with three dodecyl chains for the first time the self-assembly of the single chromophore merocyanine **1** could be investigated over a concentration range of five orders of magnitude in very unpolar solvents like MCH, where aggregation is highly favoured. By this means, the stepwise growth into dimers and a larger aggregate species was observed in solvent- and concentration-dependent UV/Vis studies. The aggregation mechanism and the structures of the emerging aggregates were studied in detail to provide an illustrative example for the formation of defined, small-sized aggregates by stepwise anti-cooperative self-assembly.

## Results and discussion

### Synthesis

**Scheme 1:**
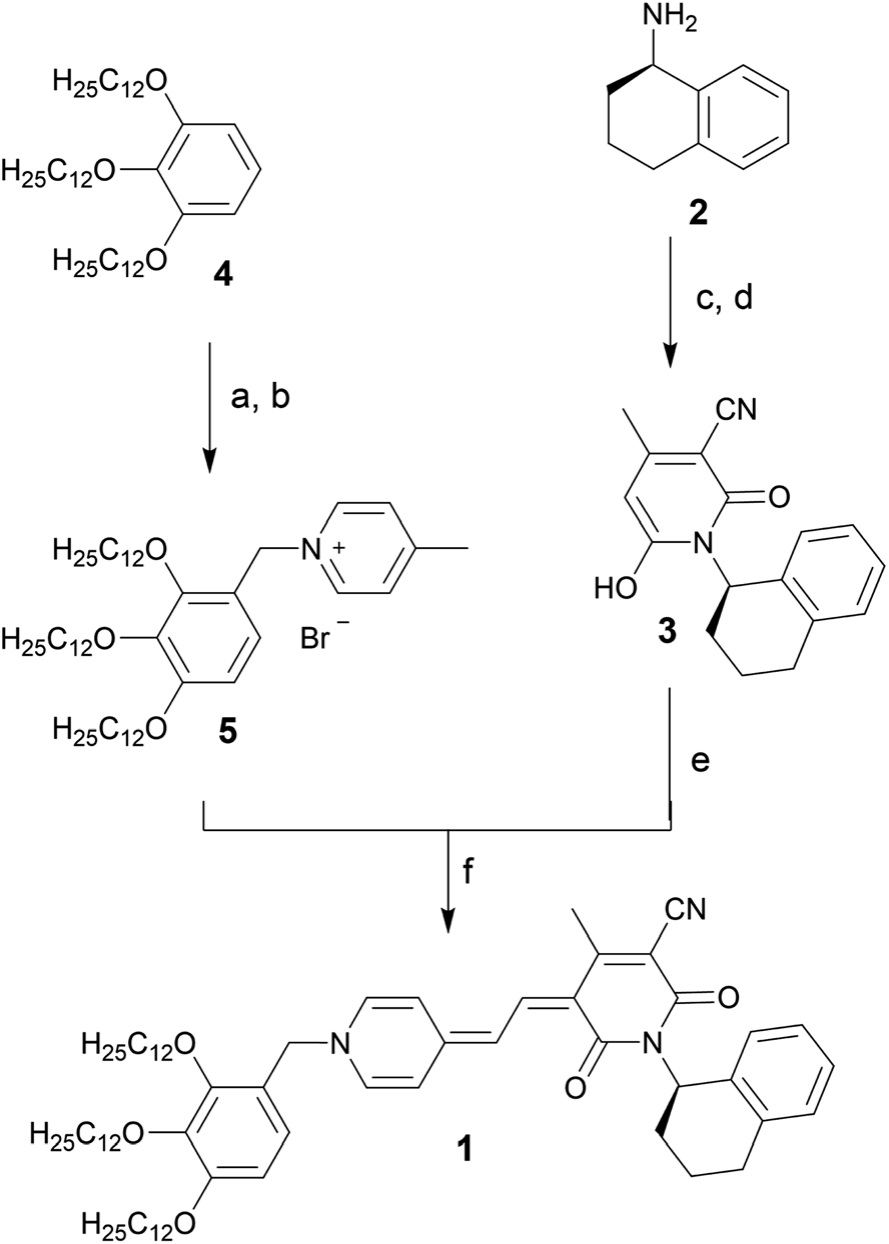
Synthesis of enantiopure merocyanine **1**. (a) HBr, (CH_2_O)_*n*_, HOAc, 70 °C, 4 h. (b) 4-Picoline, MeCN, 90 °C, 18 h, 42% over two steps. (c) NCCH_2_CO_2_Et, MeOH, reflux, 48 h. (d) AcCH_2_CO_2_Et, piperidine, 100 °C, 24 h, 21% over two steps. (e) *N*,*N*′-Diphenylformamidine, Ac_2_O, rt to 90 °C, 45 min. (f) KOAc, 100 °C, 14 h, 19% over two steps.

Merocyanine **1** was synthesized according to Scheme 1. Firstly, the enantiopure amine **2** (ee 99.3%) was used to synthesize pyridone **3** by a sequence of condensation reactions.^31^ By this means a rigid chiral center was introduced at the acceptor moiety, which due to the close proximity to the chromophore π-core should directly affect the structure of the emerging aggregates. 4-Methylpyridinium salt **5** was obtained from 1,2,3-tris(dodecyloxy)-benzene **4** according to literature known procedure.^37^ The reaction of pyridone **3** with *N*,*N′*-diphenylformamidine (DPFA) and precursor **5** gave the desired merocyanine **1** in 19% yield after purification by column chromatography. This method has been established for the synthesis of other merocyanine dyes in our earlier work.^37,38^ For details on synthetic procedures and characterization by melting point, NMR, high resolution mass spectrometry (HRMS) and elemental analysis see the ESI.†

### UV/Vis aggregation studies

**Fig. 2:**
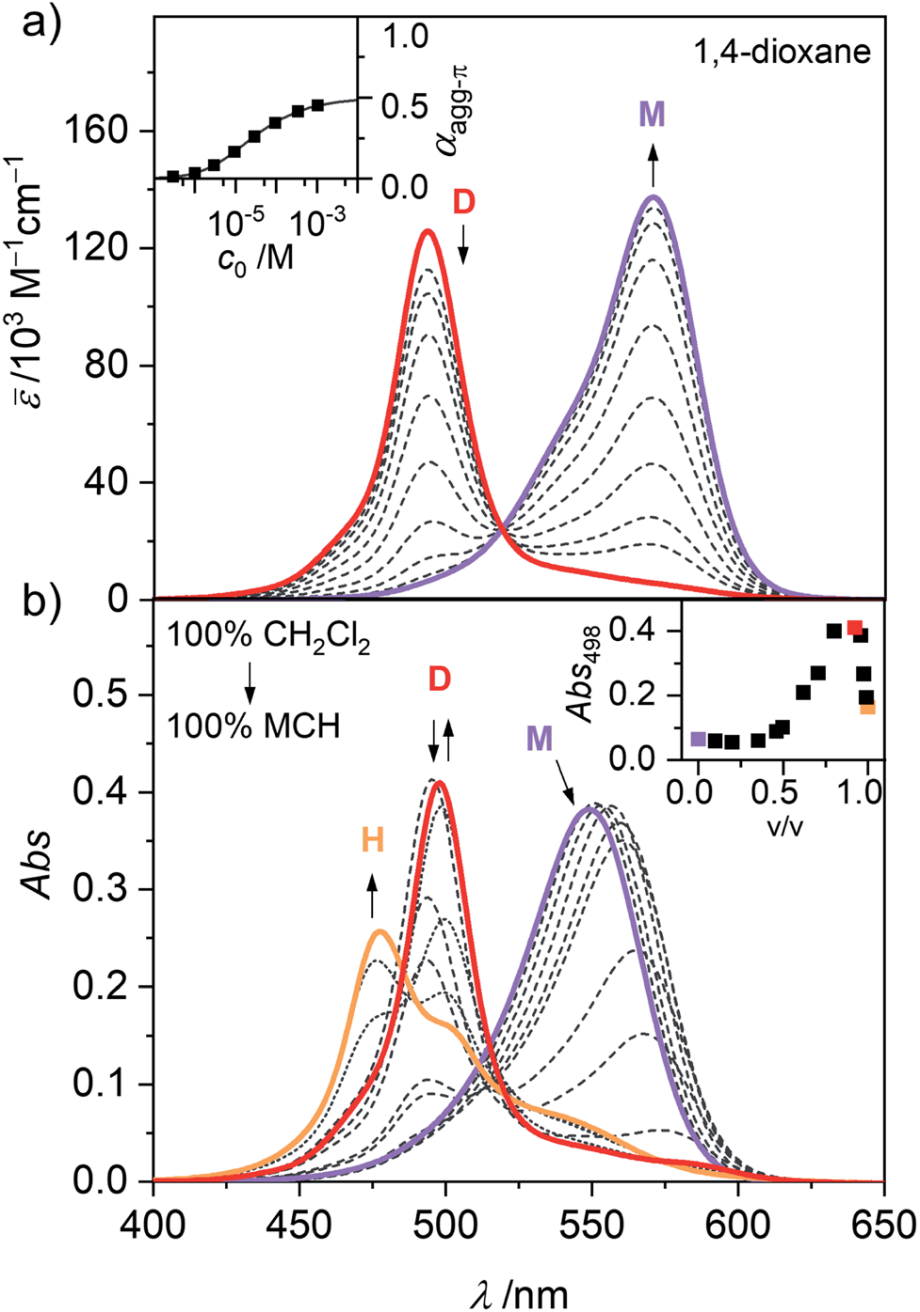
(a) Concentration-dependent UV/Vis absorption spectra (dashed grey lines) of merocyanine **1** in 1,4-dioxane at 298 K. Arrows indicate the spectral changes upon decreasing the concentration from *c*_0_ = 1.1 × 10^−3^ to 3.0 × 10^−7^ M. Colored spectra are calculated spectra for the individual monomer (M, violet) and dimer (D, red) species from global fit analysis according to the dimer model. Inset shows the concentration-dependent degree of aggregated π-faces (*α*_agg-π_) calculated from the concentration-dependent UV/Vis spectra at 571 nm (■) and the dimerization isotherm (solid line) based on the dimerization constant obtained from global fit analysis. (b) Solvent-dependent UV/Vis absorption spectra of merocyanine **1** in mixtures of CH_2_Cl_2_ and MCH (*c*_0_ = 3.2 × 10^−4^ M, 298 K). Arrows indicate spectral changes upon increasing the volume fraction of MCH from 0% to 90% (dashed lines) and to 100% (dotted lines). The spectra with a MCH content of 0% (violet), 90% (red) and 100% (orange) are marked in color. Inset shows the absorbance at 498 nm as a function of the volume fraction of MCH.

In general, the magnitude of the binding constant for a self-assembly process strongly depends on the solvent and its ability to solubilize and interact with the monomeric and aggregated species. For dipolar merocyanines, aggregation is favoured in unpolar solvents because the dipole–dipole interactions between the chromophores are stronger in unpolar compared to more polar environments. Accordingly, in the polar solvent dichloromethane (CH_2_Cl_2_), monomers prevail at all concentrations. Next, in concentration-dependent UV/Vis studies of **1** in 1,4-dioxane (Fig. 2a) only the formation of antiparallel aligned dimers (D) driven by strong dipole–dipole interactions^31^ can be observed. To trigger further aggregation of these dimer species, a solvent with even lower polarity like methylcyclohexane (MCH) is needed. In solvent-dependent UV/Vis studies (Fig. 2b) the polarity of the environment of **1** was stepwise decreased by successively changing the mixing ratio of polar CH_2_Cl_2_ and unpolar MCH. Significant spectral changes are observed as aggregate formation becomes more and more favoured.

**Fig. 3:**
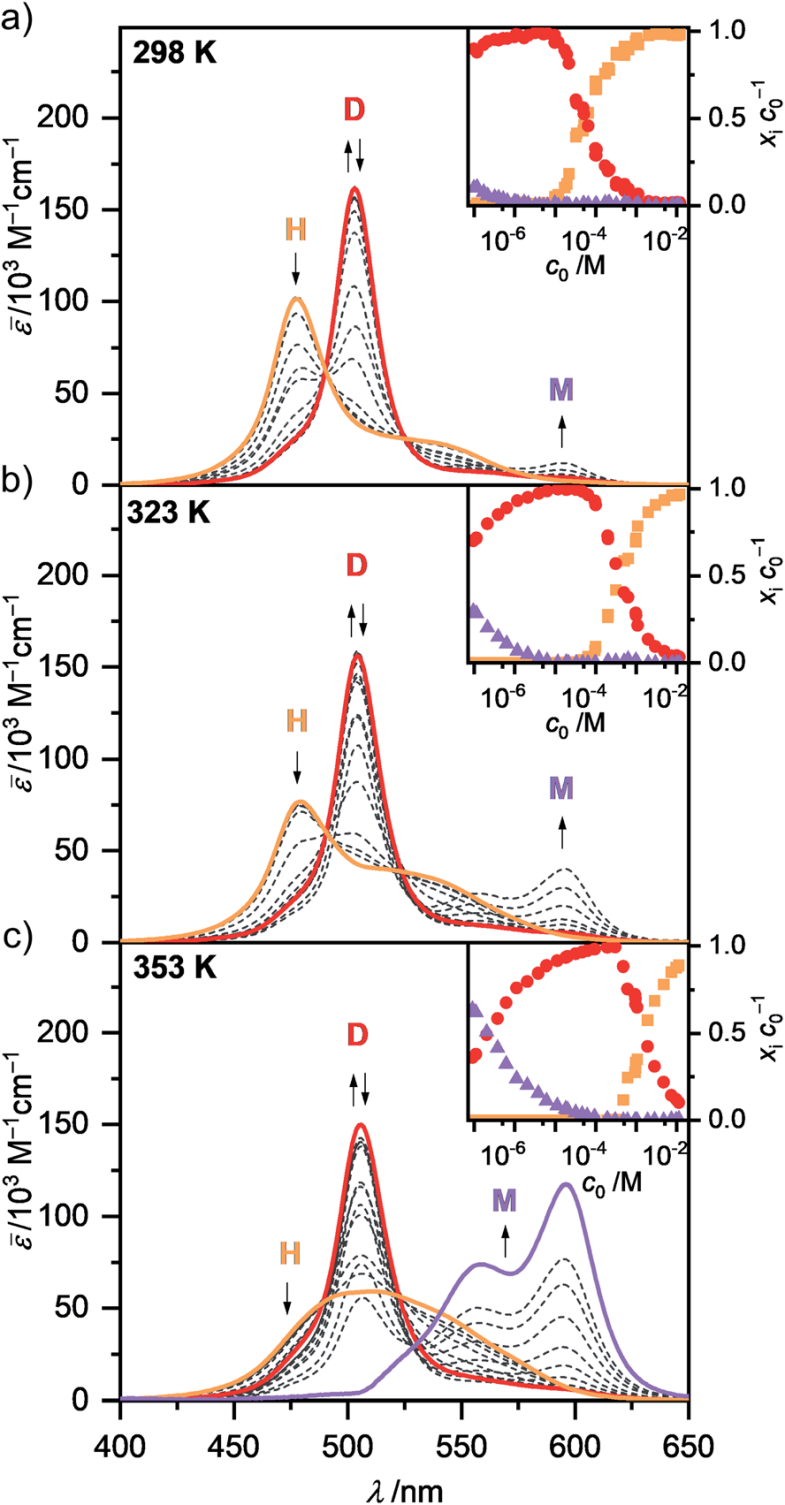
Concentration-dependent UV/Vis absorption spectra (dashed lines) of merocyanine **1** in MCH at (a) 298 K, (b) 323 K and (c) 353 K. Arrows indicate the spectral changes during the disassembly process from higher aggregates (H) to dimers (D) and monomers (M) upon decreasing the concentration from *c*_0_ = 1.0 × 10^−2^ to 9.8 × 10^−8^ M. Colored spectra are calculated spectra of the individual species from global fit analysis according to the dimer (2M ⇌ D) and pentamer (5D ⇌ H) models. Insets show the concentration-dependent fraction of molecules *x*_i_*c*_0_^−1^ of **1** present as higher aggregate (orange), dimer (red) and monomer (violet) in MCH at the respective temperatures according to multiple linear regression analysis.

In pure dichloromethane, only the monomeric species (M) is present, as evident from the strong charge transfer (CT) absorption band at *λ*_max_(CH_2_Cl_2_) = 549 nm (Fig. 2b, violet line).^31,34^ When increasing the volume fraction of MCH to about 90%, the appearance of a hypsochromically shifted absorption band, indicating H-type exciton coupling^39,40^ can be observed at *λ*_max_(CH_2_Cl_2_/MCH 1 : 9) = 498 nm (Fig. 2b, red line). These results resemble the monomer–dimer equilibrium of **1**, also observed in the concentration-dependent UV/Vis studies in 1,4-dioxane (Fig. 2a). Upon further reduction of the solvent polarity up to pure MCH an even more blue shifted absorption maximum arises at *λ*_max_(MCH) = 477 nm (Fig. 2b, orange line), which suggests the formation of an extended aggregate (H) with more than two interacting chromophores.^32,34^ For a more in depth investigation of this aggregation process, solvent-depended data unfortunately has severe disadvantages. Especially the strong solvatochromism of the push–pull chromophore^41^ (Fig. S1, ESI†) hinders in depth evaluation, due to the strong shifts and changes in the shape of the absorption band. However, the exceptionally high solubility of merocyanine **1** even in aliphatic solvents (>10 mg mL^−1^) allowed us to perform concentration-dependent UV/Vis studies in the unpolar solvent MCH over a large concentration range to investigate aggregation of this chromophore beyond the dimer species. The spectroscopic studies were performed in a concentration range of 1.0 × 10^−2^ to 9.8 × 10^−8^ M using cuvettes with an optical path length between 0.01 and 100 mm to sustain a suitable optical density. Since the chromophores tend to adsorb to glass surfaces in the low-polar MCH environment, silanized glassware (for silanization procedure see ESI†) had to be used. Furthermore, samples in MCH showed some kinetic effects and needed time to equilibrate after concentration or temperature change (Fig. S2–S3, ESI†). Accordingly, the samples were always measured several times in varying time intervals, to ensure the equilibrium state had been reached. The samples in MCH needed several hours to equilibrate after dilution and were allowed to equilibrate at room temperature overnight. After temperature change, the individual samples were measured in intervals of 5 to 10 minutes until no more changes could be observed in the respective absorption spectrum (approximately 5 to 30 min). As shown in Fig. 3, these concentration-dependent studies in MCH showed the same process as the solvent-dependent measurements.

In the unpolar solvent MCH the tendency of the dipolar merocyanine **1** to aggregate is so strong, that even at the lowest concentration accessible for UV/Vis studies (*c*_0_ = 9.8 × 10^−8^ M) almost no monomers (*λ*_max_(M) = 596 nm) are present at 298 K. However, when performing concentration-dependent measurements at elevated temperatures of 323 K and 353 K, disassembly is induced, and an increasing amount of monomer is formed in the most dilute samples (Fig. 3). The dominant peak at *λ*_max_(D) = 503 nm can be assigned to the dimeric species. Upon increasing the concentration, a further hypsochromically shifted absorption band at *λ*_max_(H) = 477 nm rises, indicating the formation of a higher aggregate species. At all temperatures a concentration range exists (*c*_0_ ∼ 6 × 10^−6^ M at 298 K, *c*_0_ ∼ 1 × 10^−5^ M at 323 K and *c*_0_ ∼ 2 × 10^−4^ M at 353 K) where almost exclusively dimer absorption can be observed. Thus, the dimer is the intermediate species in a two-step aggregation process. This preferred dimerization is expected, as the dipolar chromophores can firstly self-assemble into antiparallel dimers due to strong electrostatic dipole–dipole interactions while further aggregation into larger species is only guided by weaker dispersion forces, as the ground state dipole moment of the dimer is close to zero. Isosbestic points for the concentration-dependent spectra showing the transition from monomer to dimer, as well as from dimer to higher aggregate indicate the presence of two equilibria between each two defined species. The spectroscopic data at 353 K, showing the transition between monomer and dimer (*c*_0_ = 9.1 × 10^−8^ to 6.1 × 10^−5^ M), could be fitted globally (see ESI† for more information) according to the dimer model^14^ (Fig. S6, ESI†). A dimerization constant of *K*_D_(353 K) = 4.5 × 10^6^ M^−1^ was determined.

The concentration-dependent data showing the dimer – higher aggregate transition were best described by aggregation models for small, defined sized aggregates (trimer, tetramer and pentamer model,^32^ Fig. S7–S9, ESI†). The differences in the quality of the fit obtained for these models are small and seem to vary slightly with temperature and concentration. Together with the ill match of the fit according to the isodesmic model^14^ (Fig. S7–S9, ESI†) this is a first indication that the higher aggregate formed by merocyanine **1** is indeed not an extended π-stacked supramolecular polymer. It is possible that the size of the higher aggregates is not uniform, but smaller (trimer of dimers = hexamer) for higher temperatures and lower concentrations, where binding constants are smaller and the substituents enjoy a higher mobility, *i.e.* entropy, and larger (pentamer of dimers = decamer) for the opposite conditions. For the following evaluations, the pentamer fit is used as an approximation to describe a 5D ⇌ H equilibrium since it gave the overall best match to the experimental data. Binding constants per binding site of *K*_5_(298 K) = 6.0 × 10^4^ M^−1^, *K*_5_(323 K) = 7.5 × 10^3^ M^−1^ and *K*_5_(353 K) = 1.8 × 10^3^ M^−1^ were obtained from global fit analysis. Thus, at 353 K the aggregation constant *K*_5_ is more than three orders of magnitude smaller than the dimerization constant *K*_D_ at the same temperature, resulting in a value of *σ* > 10^3^ for the degree of anti-cooperativity. The summarized UV/Vis spectroscopic data of merocyanine **1** in CH_2_Cl_2_, 1,4-dioxane and MCH at different temperatures can be found in Table 1.

**Table 1:** Summarized UV/Vis spectroscopic data of monomer (M), dimer (D) and higher aggregate (H) species of merocyanine **1** in CH_2_Cl_2_, 1,4-dioxane (Dx) and MCH at 298 K, 323 K and 353 K after analysis of the anti-cooperative aggregation

Solvent	CH_2_Cl_2_	Dx	MCH		
*T*/K	298	298	298	323	353
*K*_D_(M–D)/M^−1^	—	4.0 × 10^4^^a^	4.0 × 10^8^^b^	4.0 × 10^7^^b^	4.5 × 10^6^^a^
*K*_5_(D–H)/M^−1^	—	—	6.0 × 10^4^^a^	7.5 × 10^3^^a^	1.8 × 10^3^^a^
*σ* = *K*_D_/*K*_5_/1	—	—	6700	5300	2500
−Δ*G*^0^(M–D)^c^/kJ mol^−1^	—	26.3	49.1	47.0	45.0
−Δ*G*^0^(D–H)^c^/kJ mol^−1^	—	—	27.3	24.0	22.0
*λ*_max_(M)^d^/nm	549	571	596	596	596
*λ*_max_(D)^d^/nm	—	494	503	504	506
*λ*_max_(H)^d^/nm	—	—	477	479	505
*ε*_max_(M)^d^/M^−1^ cm^−1^	116 000	137 000	117 000	117 000	117 000
*ε*_max_(D)^d^/M^−1^ cm^−1^	—	126 000	162 000	156 000	150 000
*ε*_max_(H)^d^/M^−1^ cm^−1^	—	—	101 000	77 000	59 000
*λ*_isosbestic_(M–D)/nm	—	519	525	528	530
*λ*_isosbestic_(D–H)/nm			489	490	489
*μ*_eg_(M)^d^/D	10.4	10.7	10.8	10.8	10.8
*μ*_eg_(D)^d^/D	—	9.5	9.5	9.7	9.7
*μ*_eg_(H)^d^/D	—	—	9.7	9.9	10.1

a Global fit analysis.

b Estimated according to dimer model from *α*_agg-π_.

c Determined from *K*_D_ or *K*_5_ by Gibbs–Helmholtz equation.

d All values correspond to monomeric units within the aggregate; for information on the determination of *μ*_eg_ see ESI.

The shape of the higher aggregate absorption band changes remarkably upon heating (Fig. 3 and S4, ESI†). While the absorption spectrum at 298 K shows a pronounced maximum at 477 nm as expected for cofacially stacked H-aggregates, the spectrum at 353 K exhibits a broad, ill-defined absorption band with a maximum at around 500 nm. These spectroscopic changes can be rationalized by a more disordered and less tight packing of the higher aggregate at higher temperatures, probably due to increased flexibility and mobility of the substituents. This shifting of the maximum of the higher aggregate band into the range of the dimer absorption indicates, that at elevated temperatures the chromophores are not packed with equal and close distances anymore but rather into preferred dimer-pairs whose contact to other dimer-pairs is less defined and/or more distant. So while at 298 K exciton coupling occurs between all the chromophores of the stack, leading to the hypsochromically shifted H-band, at 353 K the coupling between only two chromophores seems to be the most prominent. At 323 K either an intermediate higher aggregate form might be present or a mixture of the more tightly packed low and the more loosely packed high temperature form, since the UV/Vis absorption spectrum shows a sharp H-band at 479 nm as well as a more pronounced shoulder at >500 nm. The structural rearrangement in the higher aggregate structure upon heating is also characterized by an activation energy. Accordingly, time-dependent UV/Vis experiments reveal a period of about 1.5 h to return to the original room temperature (298 K) H-aggregate species (Fig. S3, ESI†). The absorption spectrum of the dimer shows only a slight broadening and red shift for increased temperatures. For the monomer no significant temperature-dependence of the shape of the absorption is observed in the experimental data, thus the calculated monomer spectrum from global fit analysis of the data at 353 K according to the dimer model is used for further evaluation of spectroscopic data at all temperatures.

Multiple linear regression (MLR) analysis was used to determine the concentration of molecules present as monomer (*x*_M_), dimer (*x*_D_) and higher aggregate (*x*_H_) in the sample solutions at different concentrations *c*_0_ (see ESI† for more information) for each data set at the applied temperature. Based on Lambert–Beer's law (eqn (1)) the extinction *E*(*λ*) can be deconvoluted into the contributions of the individual species (*ε*_M_(*λ*), *ε*_D_(*λ*) and *ε*_H_(*λ*), Fig. S10, ESI†) for each cuvette thickness (*d*).

1 *E*(*λ*) = *c*_0_*ε*(*λ*)*d* = [*x*_M_*ε*_M_(*λ*) + *x*_D_*ε*_D_(*λ*) + *x*_H_*ε*_H_(*λ*)]*d*.

The concentration-dependent fraction of molecules present as monomer, dimer or higher aggregate (*x*_i_*c*_0_^−1^) are depicted in the insets of Fig. 3. Notably, for all three temperatures a concentration range exists, where almost exclusively dimers (>99%) are present. This strict separation of the aggregation process into two consecutive steps allows us to calculate the degree of aggregated π-faces (*α*_agg-π_) independently for both equilibria, namely the dimerization (2M ⇌ D) as well as the further self-assembly (5D ⇌ H) at higher concentrations. In general, *α*_agg-π_ is defined as2
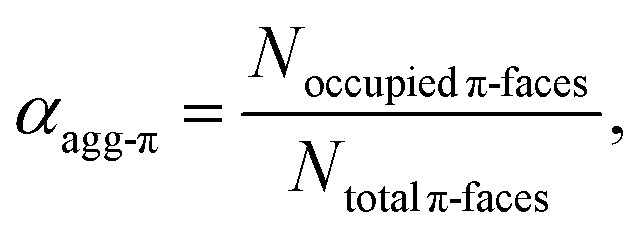
which can be applied to the two equilibria of interest (for derivation see ESI†):3
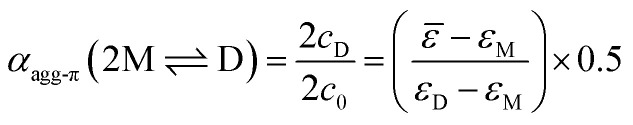
4
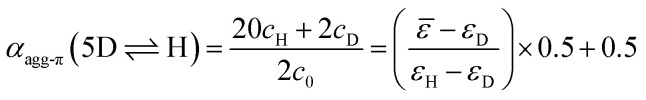


**Fig. 4:**
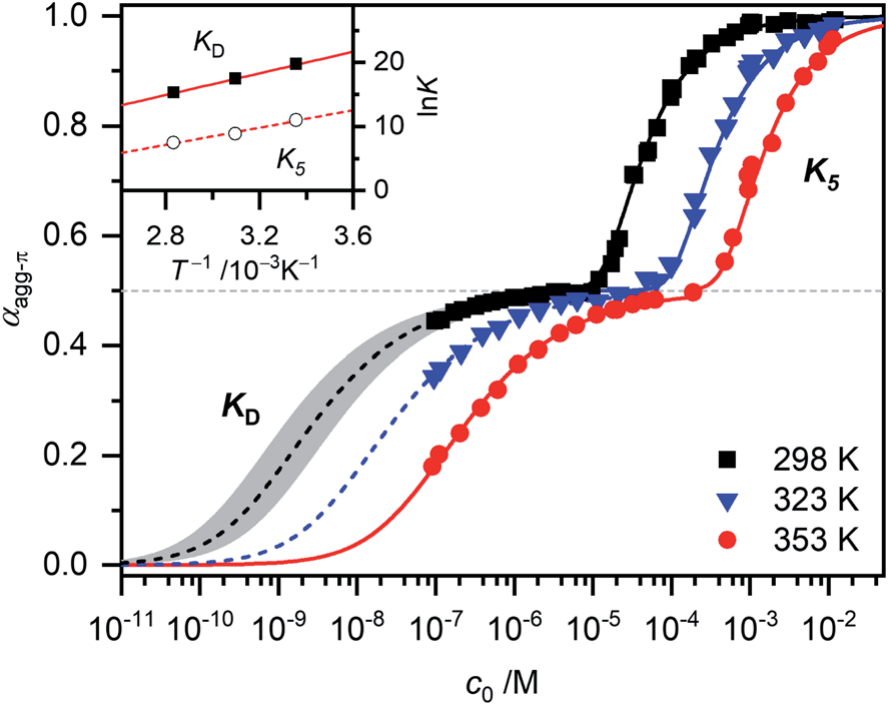
Fraction of aggregated π-faces *α*_agg-π_ of merocyanine **1** in MCH calculated from spectroscopic data at 298 K (black symbols), 323 K (blue symbols) and 353 K (red symbols) according to eqn (3) and (4). Simulated curves according to dimer (*α*_agg-π_ < 0.5, eqn (5)) and pentamer model (*α*_agg-π_ > 0.5, eqn (6) and (7)) calculated with binding constants from global fit analysis (solid lines) or estimated binding constants (dashed lines) are shown for comparison. Grey area marks a range of *α*_agg-π_ for 9.0 × 10^8^ M^−1^ > *K*_D_ > 2.0 × 10^8^ M^−1^. Inset shows the van't Hoff plot for the calculation of thermodynamic parameters for dimerization (*K*_D_) and formation of the higher aggregate (*K*_5_).

For simplification, it is assumed that for the higher aggregate species all π-faces are occupied so *α*_agg-π_ = 1 is defined for the fully aggregated state. Please note, that this would indeed only be true for a cyclic arrangement of molecules or an infinite stack. The experimental apparent extinction coefficient *ε̄* of a sample with the concentration *c*_0_ is taken at the maximum of the dimer (5D ⇌ H) or the monomer (2M ⇌ D) band. The monomer (*ε*_M_), dimer (*ε*_D_) and higher aggregate (*ε*_H_) extinction coefficients are obtained from the spectra of the individual species calculated by global fit analysis. Up to the concentration at which the absorption band of the dimer reaches its maximum, *α*_agg-π_ was calculated according to eqn (3), above that eqn (4) was used. The data points from the concentration-dependent UV/Vis studies at 298, 323, and 353 K nicely follow the trend expected for anti-cooperative aggregation behaviour schematically illustrated in Fig. 1, with a plateau at *α*_agg-π_ = 0.5 where predominantly the dimer species is present (Fig. 4).

The experimental data for the initial dimerization of merocyanine **1** at 353 K nicely match the theoretical curve for *α*_agg-π_ according to the dimer model^14^ (eqn (5)) with *K*_D_(353 K) = 4.5 × 10^6^ M^−1^ from global fit analysis.5
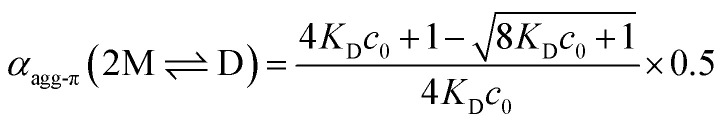


Although *α*_agg-π_ only decreases to a value of 0.43 and 0.34 for the measurements at 298 K and 323 K, respectively, the dimerization constants can be estimated by comparing simulated curves for different *K*_D_-values with the experimental data points. The dimerization constant at 298 K can thus be assessed to be around *K*_D_(298 K) = 4.0 × 10^8^ M^−1^ (Fig. 4, black dashed line). For comparison, the gray area in Fig. 4 marks a range of *α*_agg-π_ for 9.0 × 10^8^ M^−1^ > *K*_D_ > 2.0 × 10^8^ M^−1^. In the same way the binding constant at 323 K was estimated to *K*_D_(353 K) = 4.0 × 10^7^ M^−1^, which is in accordance to the decrease in *K*_D_ expected for increasing temperatures. Furthermore, the simulated curves for the 5D ⇌ H equilibrium according to the pentamer model are shown in Fig. 4. Since the pentamer model consists of a fifth-order equation, no simple expression can be given for *α*_agg-π_. However, the degree of aggregated π-faces can be simulated according to eqn (6) (for derivation see ESI†).6
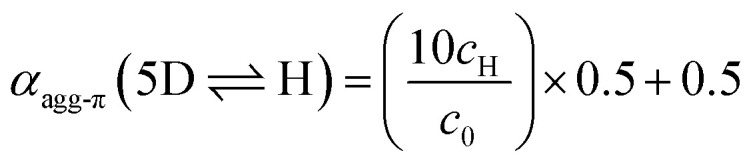


The concentration of higher aggregate *c*_H_ can be calculated for different total molecular concentrations *c*_0_ by finding the zero-crossings of eqn (7).7
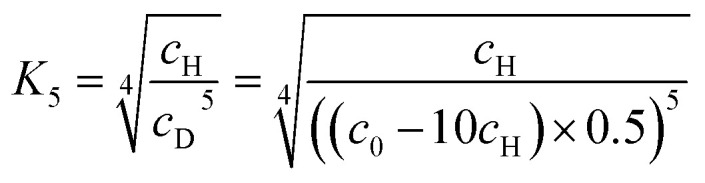


With the binding constants for dimerization and further aggregation, the cooperativity parameter *σ* = *K*_D_/*K*_5_ of the self-assembly process of merocyanine **1** in MCH can be calculated to: *σ* (298 K) = 6700, *σ* (323 K) = 5300 and *σ* (353 K) = 2500. It quantifies the high degree of anti-cooperativity in this system.

In order to determine the thermodynamic parameters for the dimerization (*K*_D_) and aggregation (*K*_5_) process, the binding constants at the three different temperatures were used in a van't Hoff plot (Fig. 4, inset). The values for both *K*_D_ and *K*_5_ meet the expected linear relation for ln *K* and *T*^−1^. However, the results of the van't Hoff plot for *K*_5_ should be interpreted with caution, since the structure of the higher aggregate might be different at elevated temperatures (*vide supra*). Thus, it is not certain that a reasonable comparability is given for the *K*_5_ values at different temperatures. The determined entropy penalty for the formation of the higher aggregate (Δ*S*^0^ = −96.4 J K^−1^ mol^−1^) is higher than for the dimerization (Δ*S*^0^ = −74.4 J K^−1^ mol^−1^), since more building blocks are involved in the process. As expected, dimerization is enthalpically more favoured (Δ*H*^0^ = −71.5 kJ mol^−1^) than the formation of the higher aggregate (Δ*H*^0^ = −55.7 kJ mol^−1^), which can be explained by the strong dipole–dipole interaction within the dimer. The Gibbs free energy changes Δ*G*^0^ obtained from this analysis for dimer (Δ*G*^0^(353 K) = −45.2 kJ mol^−1^) and higher aggregate (Δ*G*^0^(298 K) = −27.0 kJ mol^−1^) formation match the values calculated from the binding constants from global fit analysis at the respective temperatures (Table 1) very well. The highly negative Δ*G*^0^ values again confirm the exceptionally strong binding of the dipolar merocyanine chromophores in MCH compared to other solvents.^31^

### Investigation of the aggregate structures

**Fig. 5:**
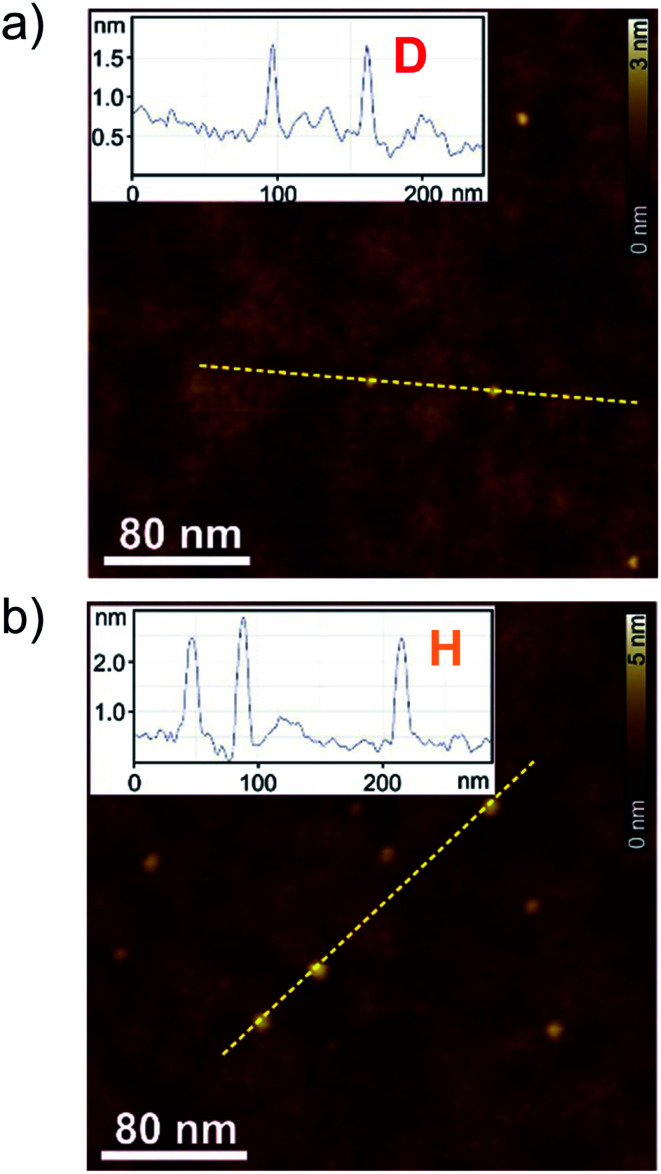
AFM height images of (a) dimer samples of merocyanine **1** in MCH (*c*_0_ = 8.7 × 10^−6^ M) and (b) higher aggregate sample of merocyanine **1** in MCH diluted from *c*_0_ = 9.7 × 10^−4^ M to *c*_0_ = 0.5 × 10^−4^ M and subsequent spin-coating onto TPA-functionalized SiO_*x*_/AlO_*x*_ substrates. Insets show the respective cross-section analysis of the yellow dashed lines.

To correlate the spectroscopic findings as well as the investigated anti-cooperative growth mechanism with structural parameters it is essential to deduce the number of molecules which form the aggregate species as well as the respective molecular arrangement within the defined assembly. The formation of a small-sized higher aggregate species indicated by the fitting results of the spectroscopic data was confirmed by AFM, DOSY and VPO measurements. The higher aggregate sample for AFM imaging was prepared by diluting a MCH solution of merocyanine **1** at a concentration of *c*_0_ = 9.7 × 10^−4^ M to 0.5 × 10^−4^ M, and immediately spin-coating it onto silicon wafer functionalized with *n*-tetradecylphosphonic acid (TPA). Time-dependent UV/Vis studies have shown that due to kinetic effects the higher aggregate is still the dominant species for at least ten minutes after dilution at 298 K (Fig. S2, ESI†). The AFM images show very uniform small particles of 2.3 ± 0.2 nm height and 7–9 nm diameter (Fig. 5b). Also for dimer-samples small homogeneous particles were observed (Fig. 5a). The respective height of 0.9 ± 0.2 nm matches well the height of two π-stacked molecules on the substrate surface. The average diameter is measured to be 4.5 nm, however, this value is not reliable since the diameter of objects smaller than the tip radius (<7 nm) is overestimated in AFM measurements due to the tip-broadening effect.^42,43^

VPO measurements were performed in MCH at 318 K to determine the average molar mass of the higher aggregate and thus the average number of molecules incorporated in those aggregates (for detailed information see ESI†). For sample concentrations >4 g kg^−1^ (corresponding to 3 × 10^−3^ M) an average number of six molecules (*i.e.* three dimers) per higher aggregate particle was deduced by this method, using benzil as well as polystyrene PS5279 as reference. This value is at the lower end of the range expected for the higher aggregate size according to the fitting results of the UV/Vis data (*vide supra*), but considering the error of the method and the elevated temperatures needed for the measurements it is still supporting our previous assumptions.

^1^H DOSY measurements of the higher aggregate in MCH-*d*_14_ (*c*_0_ = 1.0 × 10^−3^ M, 295 K) emphasize the uniformity of the aggregate particles with a hydrodynamic radius of 2.4 nm according to the Stokes–Einstein equation. This is in good agreement with the particle size observed by AFM. The hydrodynamic radius matches the size of a geometry-optimized stack of ten merocyanine **1** chromophores (*i.e.* five dimers) considering that the large solubilizing substituents were neglected in the calculations (Fig. S22, ESI†). For the dimer (MCH-*d*_14_, *c*_0_ = 2.3 × 10^−4^ M, 348 K) a hydrodynamic radius of 1.0 nm was calculated from the diffusion coefficient. Also this value agrees well, with the size of the geometry optimized dimer-structure of **1** (Fig. S23, ESI†).

**Fig. 6:**
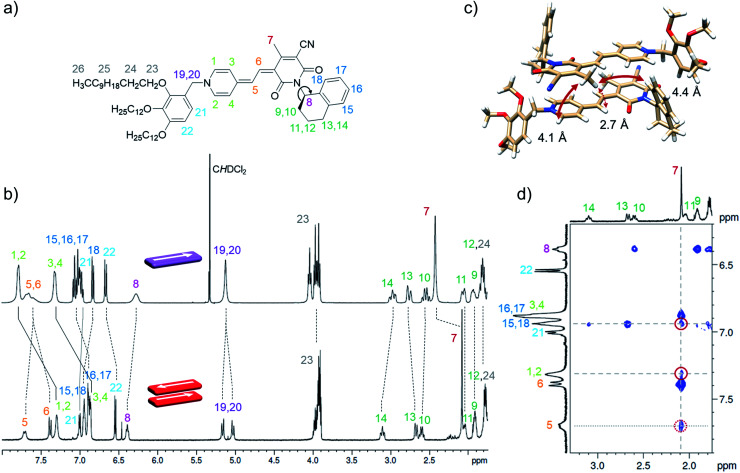
(a) Molecular structure of merocyanine **1** with proton assignment in color. (b) Relevant section of the ^1^H NMR spectra of the monomer (top) of **1** in CD_2_Cl_2_ at 295 K (*c*_0_ = 9.7 × 10^−4^ M, 400 MHz) and the dimer (bottom) in MCH-*d*_14_ at 348 K (*c*_0_ = 2.2 × 10^−4^ M, 600 MHz). (c) Geometry-optimized dimer structure (B97D3/def2-SVP) with distances given for protons showing intermolecular NOE cross peaks with the protons 7 of the CH_3_ group. (d) Section of the ^1^H ^1^H NOESY NMR (600 MHz) spectrum of the dimer of **1** (*c*_0_ = 2.7 × 10^−4^ M) in MCH-*d*_14_ at 348 K. For complete spectrum see ESI.†

As the global fitting of the spectroscopic data as well as AFM, VPO and DOSY measurements all indicate the formation of a small-sized oligomeric higher aggregate consisting of six to ten molecules, we took the efforts to get a better understanding of the arrangement of the chromophores within this species. To elucidate the structure of the dimer and the higher aggregate formed by merocyanine **1** in MCH, 1D and 2D NMR spectra were recorded (see ESI†). All peaks in the ^1^H NMR spectra of the monomer and dimer could be assigned to the respective protons with the help of correlation spectroscopy (COSY), nuclear Overhauser effect spectroscopy or rotating frame Overhauser effect spectroscopy (NOESY or ROESY), and heteronuclear single quantum coherence (HSQC) experiments (Table S2, ESI†). The proton spectrum of the dimer (MCH-*d*_14_, *c*_0_ = 2.2 × 10^−4^ M, 348 K) is well resolved and shows only one set of signals revealing a high symmetry of the dimer structure with two equivalent chromophores (Fig. 6b).

In comparison to the monomer ^1^H spectrum (Fig. 6b, CD_2_Cl_2_, *c*_0_ = 9.7 × 10^−4^ M, 295 K), only the signal for the two protons 19 and 20 splits in the dimer spectrum as these protons are now closer to a stereogenic centre and the difference in the chemical environment of those diastereotopic protons is increased within the dimer structure compared to the monomeric species. For the proton 8 at the chiral carbon, only one signal can be observed for the monomer as well as for the dimer. In principle the chiral tetralin substituent can point toward the interior or exterior of the double π-stack with proton 8 pointing either towards the outer or the inner neighbouring carbonyl group. As only one signal can be observed for proton 8 in the monomer as well as in the dimer ^1^H spectrum, fast rotation around the marked C–N bond Fig. 6a is expected for both cases. This is further augmented by a calculated rotational barrier around this C–N bond of only 52 kJ mol^−1^ (B97D3/def2-SVP) for the monomer in the gas phase, leading to a half-life of *τ*_1/2_(298 K) = 2.1 × 10^−4^ s, which is fast on the NMR timescale of this process.

The defined molecular arrangement of the dipolar chromophores within the dimer could be further elucidated by NOE spectroscopy. In the dimer ^1^H ^1^H NOESY NMR spectrum (MCH-*d*_14_, *c*_0_ = 2.7 × 10^−4^ M, 348 K) several cross signals can be found, where no signals are expected for the monomer, indicating intermolecular proximity of the protons. Especially the sharp proton signal of the CH_3_-group 7 of the acceptor unit is well suited to detect those intermolecular proximities and gives defined NOE cross peaks with the outer pyridine protons (1, 2) and the aromatic proton 18 of the tetralin substituent (Fig. 6d). Notably, these cross signals are not observed in the spectrum of the monomer (Fig. S17, ESI†), which corroborates the presence of intermolecular through space couplings. The results are in accordance with the geometry-optimized structure (Fig. 6c) indicating close spatial proximity between the mentioned protons. Additionally, cross signals can be observed between the protons 7 and both methine bridge protons (5, 6). Even though the intramolecular distance between 7 and 5 is with 4.9 Å also still just within the NOE range the much closer intermolecular contact between those protons can explain the relatively high intensity of the cross signal observed between those protons (Fig. 6d). A similar intermolecular arrangement has also been observed in the crystal structure of the same merocyanine chromophore equipped with other substituents.^31^

**Fig. 7:**
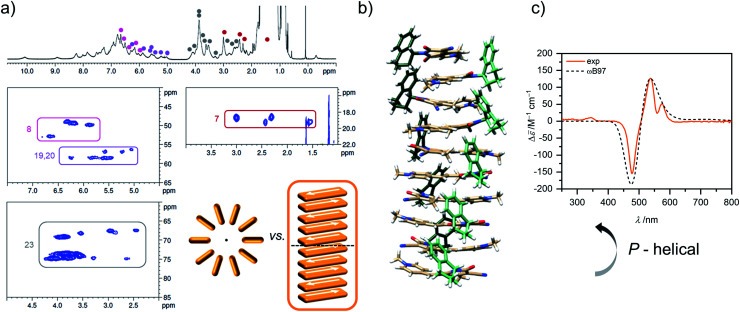
(a) ^1^H as well as sections of the ^1^H ^13^C HSQC NMR (600 MHz) spectrum (for complete spectrum see Fig. S21, ESI†) of the higher aggregate of **1** (*c*_0_ = 2.1 × 10^−3^ M) in MCH-*d*_14_ at 295 K. Signals of protons with characteristic ^13^C shift could be assigned in the HSQC spectrum of the higher aggregate and are marked in color. Sketch of cyclic *vs.* linear arrangement of ten chromophores visualizes the lower degree of symmetry of the linear stack. (b) Geometry-optimized structure of a decamer stack of **1** (PM7, trialkoxypheny substituents replaced by methyl groups after structure optimization to reduce computational effort for the TDDFT calculations). (c) Experimental CD spectrum of the higher aggregate of **1** in MCH (orange, *c*_0_ = 1.0 × 10^−3^ M, 298 K) in comparison with calculated CD spectrum (dashed grey) of the structure shown in (b) calculated by TDDFT with the ωB97 functional (def2SVP, PCM, 15 states, half with at half height = 0.18 eV, shifted 0.64 eV toward lower energies and intensity corrected to fit the maximum of the experimental spectrum).

Unfortunately, the proton spectrum of the higher aggregate in MCH-*d*_14_ at 295 K shows broad peaks (Fig. 7a), which prohibits an unambiguous proton assignment like for the dimer species. Still, some information can be deduced from the HSQC spectrum of the higher aggregate. Since ^13^C shifts are in general hardly influenced by aggregation (Table S2, ESI†), some proton signals can be assigned in the higher aggregate ^1^H spectrum based on the HSQC spectrum (Fig. 7a).

This works especially well for the protons 7 (*δ*_C_(D) = 18.2 ppm), 8 (*δ*_C_(D) = 49.9 ppm), 19/20 (*δ*_C_(D) = 58.9 ppm), and 23 (*δ*_C_(D) = 69.6, 73.9, 74.2 ppm) as the corresponding carbon atoms have a characteristic shift in comparison to the other carbons of the molecules. Notably, defined sets of cross signals can be observed in the HSQC of the higher aggregate. For the protons 7 of the methyl group at least four signals (red labels in Fig. 7a) can be assigned and also for the other protons (labelled in different colors in Fig. 7a) several sets of signals are observed. The signal sets cover a wide range of about 1.5 ppm, indicating large variation in the electronic environment of the chromophores within the higher aggregate. This condition is realized in a linear stack of chromophores, where the environment of the outer molecules differs significantly from that of the inner ones. In contrast, a cyclic structure does not seem reasonable since due to the higher symmetry less signals are expected (illustration in Fig. 7a). For our structure proposal of the higher aggregate the linear arrangement is therefore favoured over a cyclic arrangement of chromophores. The limited size of the stack to presumably a decamer (*vide supra*) can be explained by the significant sterical demand of the peripheral solubilizing trialkoxyphenyl brushes, which cannot be accommodated within the close repeat distance, demanded by the π-stacked dimers, hindering the further growth to an extended assembly (Fig. S28, ESI†). These sterical effects^24,25,27,28^ in combination with the preferred formation of small-sized aggregates by the anti-cooperative aggregation mechanism^19,20,26^ can well explain the formation of oligomers of rather defined-size instead of extended π-stacks.

Additional information on the relative arrangement of chromophores within an aggregate can be gained from CD spectroscopy. While the CD signals of the monomer and the dimer of **1** are very weak (Fig. S24, ESI†), the CD spectrum of the higher aggregate shows a much stronger bisignate signal with positive Cotton effect (Fig. 7c), indicating a *P*-helical arrangement of the chromophores according to the exciton chirality method.^44^ The CD band exhibits two maxima at *λ* = 575 nm and *λ* = 537 nm in the wavelength range of the shoulder of the higher aggregate absorption band and a minimum at *λ* = 477 nm which coinsides well with the absorption maximum. The simulated CD spectrum by time-dependent density functional theory (TDDFT) calculations on the geometry-optimized decamer stack with *P*-helicity (Fig. 7b) indeed reproduces the experimental spectrum quite well. This gives further evidence that our proposed structure for the higher aggregate is a reasonable approximation for the true arrangement in solution. Note that vibronic coupling is not considered by our employed TDDFT method so that the vibronic fine structure at *λ* > 500 nm is not reproduced by the simulations.

### Aggregation-induced emission enhancement

Aggregation-induced emission (AIE)^45^ and aggregation-induced emission enhancement (AIEE)^46^ for organic nanoparticles became popular research fields during the last two decades. Both terms describe a related phenomenon, *i.e.* an increase of luminescence upon aggregation. Whilst the original example for which the term AIEE was coined described an increase of fluorescence with the formation of nanoparticles composed of π-stacked “aggregated” dyes (similar as in Scheibe's J-aggregates^47^) with relevance for the design of luminescent organic semiconductors,^4,48^ no π–π-stacking was involved in nanoparticles of 1-methyl-1,2,3,4,5-pentaphenylsilole dye for which the term AIE was coined.^45^ Indeed, for these aggregates the fluorescence enhancement did not originate from dye–dye interactions upon aggregation but from the restriction of motions that promote the non-radiative decay.^49^ Like for AIEE also for this phenomenon of AIE multiple examples from old literature were already available including fluorescence enhancements in viscous media, upon cooling, upon solidification or by fixation of the π-scaffold with covalent bridges.^50^ However, because both of these as well as the majority of all other known AIE and AIEE molecules only allow the investigation of either the molecular state or the often poorly characterized nanoparticle state, the latter often consisting of thousands of molecules and including heterogeneity, few insights are provided by these studies on the origin of the fluorescence enhancement upon dye aggregation. In this regard the dimers and decamers of merocyanine **1** offer a unique opportunity to acquire further insights into the emergence of fluorescence beyond previously studied merocyanine dimers^51^ and solid state materials.^52^ The fluorescence properties of the monomer, dimer and higher aggregate species were investigated and the summarized results can be found in Table 2.

**Table 2:** Summarized fluorescence data of monomer (M), dimer (D) and higher aggregate (H) of merocyanine **1** in solution and in the solid state (A, B) at room temperature

	Species	*λ*_em_/nm	Δ*ν̄*_Stokes_/cm^−1^	*τ*^a^/ns	*Φ*_Fl_/%	*k*_r_/10^6^ s^−1^	*k*_nr_/10^6^ s^−1^
CH_2_Cl_2_	M^b^	587	1200	<0.2	0.23	>11.5^g^	>5000^g^
MCH	D^c^	720	6000	7.6	2.3	3.0	129
	H^d^	706	6800	11.5	4.5	3.9	83.1
Solid	A^e^	710	—	2.3 (27%), 8.1 (73%)	4.9	—h	—h
	B^f^	710	—	1.7 (28%), 5.1 (72%)	7.5	—h	—h

a Decay curves can be found in Fig. S25, ESI.

b *c*_0_ = 7 × 10^−7^ M, OD = 0.08.

c *c*_0_ = 2 × 10^−6^ M, OD = 0.25; it was verified with a more dilute sample (OD < 0.03) that excitation spectrum is free of reabsorption effects.

d *c*_0_ = 1 × 10^−3^ M, OD = 0.9, front face setup.

e Freeze-dried higher aggregate solution of **1** in cyclohexane (*c*_0_ = 1 × 10^−3^ M).

f Solid after evaporation of CH_2_Cl_2_ and drying *in vacuo*.

g Only a lower limit for *k*_r_ and *k*_nr_ is given, as the fluorescence lifetime is below the instrument response time of the TCSPC setup.

h Not evaluated because the biexponential decay suggests the presence of a mixture of monomer-like and aggregate species.

**Fig. 8:**
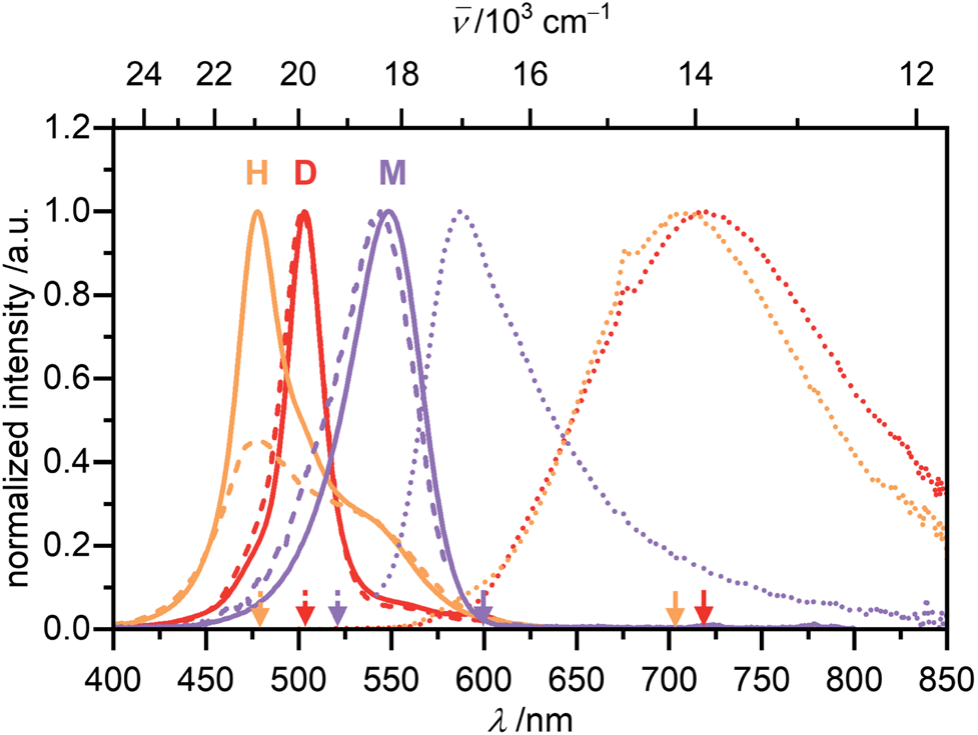
Normalized UV/Vis absorption (solid line), fluorescence (dotted line) and excitation (dashed line) spectra of the monomer (violet, *c*_0_ = 6.6 × 10^−7^ M, *λ*_ex_ = 520 nm, *λ*_em_ = 600 nm) of **1** in CH_2_Cl_2_, as well as the dimer (red, *c*_0_ = 1.7 × 10^−6^ M, *λ*_ex_ = 502 nm, *λ*_em_ = 720 nm) and the higher aggregate (orange, *c*_0_ = 1.0 × 10^−3^ M, *λ*_ex_ = 478 nm, *λ*_em_ = 703 nm, front face setup) of **1** in MCH all recorded at room temperature.

Monomeric merocyanine dyes usually show only poor fluorescence due to a fast non-radiative deactivation pathway through a bond-twisting mechanism (*Φ*_Fl_(M) ∼ 0.1%).^53–56^ Rigidification of the π-system by dimerization,^51^ stacking^34^ or in the solid state^52^ can enhance the emission strength and accordingly merocyanines were also utilized in aggregation-induced emission studies.^57^ The monomer emission of **1** could not be investigated in MCH since low sample concentrations of <10^−7^ M are required to obtain significant amount of monomer, which however resulted in no detectable monomer emission at elevated temperatures of 353 K, where a monomer content of >65% can be reached. The fluorescence properties of the monomer where therefore studied in CH_2_Cl_2_, where at all concentrations suitable for spectroscopy exclusively the monomeric species is present (Fig. 8, violet).

The monomer's emission spectrum with maximum at *λ*_em_(M) = 587 nm is mirror-imaged to the monomer absorption with a Stokes shift (Δ*ν̃*_Stokes_) of 1200 cm^−1^. As expected, a very short lifetime (*τ* < 0.2 ns) and a low quantum yield of *Φ*_Fl_(M) = 0.23% could be determined for the monomer species. The dimer of **1** in MCH shows a structureless broad excimer emission band at *λ*_em_ = 720 nm (*λ*_ex_ = 502 nm, *c*_0_ = 1.7 × 10^−6^ M, 295 K) with large Δ*ν̃*_Stokes_ = 6000 cm^−1^ (Fig. 8, red) and a lifetime of the excited state of 7.6 ns. Even though the dimer is more emissive than the monomer the fluorescence intensity is still comparably low. Accordingly, a quantum yield of *Φ*_Fl_(D) = 2.3% could be determined for the dimer in MCH. Interestingly, for the higher aggregate of merocyanine **1** in MCH an even further increased fluorescence intensity and lifetime of *τ* = 11.5 ns were observed compared to the dimer. The fluorescence spectrum of the higher aggregate also shows similar to the dimer a broad excimer band at *λ*_em_(H) = 706 nm (*λ*_ex_ = 478 nm, *c*_0_ = 1.0 × 10^−3^ M, 295 K) with an even larger Δ*ν̃*_Stokes_ of 6800 cm^−1^ (Fig. 8, orange). The emission spectrum resembles the dimer emission, which might indicate a localization of the initially formed exciton into excited dimers (excimers).^58^ Due to the high sample concentration, the measurements suffer from reabsorption effects, even though a front face setup was used to keep the sample thickness as small as possible. These effects are less pronounced in the emission spectrum, due to the large Δ*ν̃*_Stokes_, but clearly visible in the excitation spectrum of the higher aggregate. To determine the quantum yield of the higher aggregate, the kinetic stability of the aggregate upon dilution was exploited. A concentrated higher aggregate solution of merocyanine **1** (*c*_0_ = 1.0 × 10^−3^ M) was diluted to a concentration where predominantly dimers are present under equilibrium conditions at room temperature (*c*_0_ = 1.0 × 10^−5^ M). The spectral changes of the emission were monitored over time (Fig. S26, ESI†). Excitation at the wavelength of the isosbestic point (*λ*_ex_ = 489 nm) guarantees, that the amount of photons absorbed by the sample stays constant. Upon disassembly into dimers, the fluorescence intensity decreases and the emission maximum shifts to 719 nm. The quantum yield of the higher aggregate was then estimated relative to the quantum yield of the dimer by comparing the integrated emission spectra of the first spectrum after 1 min, where according to our data obtained from UV/Vis studies 27% of dimer and 73% of higher aggregate are present, and the last spectrum at 180 min, which is 97% dimer and 3% higher aggregate. By this method a quantum yield of about *Φ*_Fl_(H) = 4.5% was determined for the H-aggregated dye stack (see ESI† for more information).

With the available data for fluorescence quantum yields and fluorescence lifetimes for the monomer and the two aggregate species, further insights into the radiative (*k*_r_) and non-radiative (*k*_nr_) decay rates could be obtained (Table 2). These data clearly indicate that the rigidification of the dyes within the aggregate state affords a pronounced decrease of the non-radiative rate whilst the reduction of the radiative rate due to H-type coupling has a weaker impact. In this regard merocyanine **1** dimer and oligomer aggregates might be considered as soluble relatives to *para*-distyrylbenzene-based emissive solid state H-aggregates.^48^

Merocyanine **1** was also found to be emissive in the solid state. Two different solid samples were investigated: a solution of the higher aggregate of **1** in cyclohexane (*c*_0_ = 1 × 10^−3^ M, Fig. S5, ESI†) was freeze-dried and for the resulting solid an absolute quantum yield of *Φ*_Fl_ = 4.9%, similar to the quantum yield of the higher aggregate in MCH, was determined with the integration sphere. A solid sample of **1**, obtained by evaporation of a CH_2_Cl_2_ solution, gave an even larger absolute quantum yield of *Φ*_Fl_ = 7.5%. Both solid samples show similar broad excimer like emission spectra with maxima at *λ*_em_ = 710 nm (Fig. S27, ESI†). Also in the solid state a significant decrease of the non-radiative rate due to rigidification seems to be the main reason for the increased fluorescence intensity compared to the monomeric molecule in solution.

### Aggregation-induced CPL enhancement

**Fig. 9:**
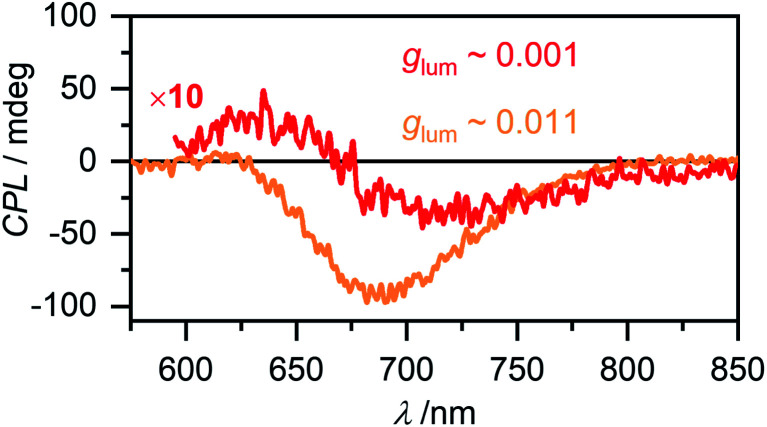
CPL spectra of the dimer (red, *c*_0_ = 6.7 × 10^−6^ M, *λ*_ex_ = 480 nm) and the higher aggregate (orange, *c*_0_ = 1.2 × 10^−3^ M, *λ*_ex_ = 470 nm) of merocyanine **1** in MCH recorded at room temperature. The dimer spectrum is depicted with ten-fold increased intensity.

Circular polarized luminescence (CPL) describes the phenomenon of differential emission intensity of right and left circularly polarized light. While CD spectroscopy provides insights about the ground state of chiral systems, CPL can grant information about the relaxed excited state. The extend of chiral fluorescence dissymmetry is quantified by the anisotropy factor *g*_lum_ = 2(*I*_L_ − *I*_R_)/(*I*_L_ + *I*_R_), with *I*_L_ and *I*_R_ being the intensities of left and right circularly polarized light, respectively. Molecular organic systems usually exhibit relatively low *g*_lum_ values of 10^−4^ to 10^−2^.^59^ It has, however, been observed that chiral excimer formation^60^ or in general the formation of chiral aggregated structures^61,62^ can enhance the *g*_lum_ value of a system significantly. Intrigued by these reports in literature, we investigated the CPL properties of the aggregate species of merocyanine **1**. For the monomer of **1** as expected no CPL was detectable, due to the low fluorescence intensity and the very week CD signal (Fig. S24, ESI†). For the dimer in MCH (*c*_0_ = 6.7 × 10^−6^ M) a CPL signal with negative Cotton effect is detected (Fig. 9, red line) with a *g*_lum_ value of 0.001 at 720 nm. The CPL spectrum of the higher aggregate in MCH (*c*_0_ = 1.2 × 10^−3^ M, Fig. 9, orange line) exhibits a strong negative signal with a significantly increased *g*_lum_ of up to 0.011 at 700 nm. Such preferential right circularly polarized fluorescence (negative CPL signal) for a right-handed excimer configuration, as proposed for our higher aggregate, has also been observed for pyrene excimers of right-handed screw sense.^63^ The *g*_lum_ value of the higher aggregate of **1** is in the same order as the highest values reported on multi-chromophore aggregates in solution^61^ and confirms the usefulness of chiral helical assemblies to enhance the efficiency of CPL by AIEE.

## Conclusion

Solvent- and concentration-dependent UV/Vis studies of merocyanine **1** revealed an anti-cooperative aggregation mechanism that consists of a two-step process. In the first step a dimer (2M ⇌ D) is formed due to strong dipole–dipole interactions followed by further self-assembly of those dimers into a larger oligomer π-stack by weaker dispersion forces. Due to the pronounced difference in the thermodynamic driving force for dimerization *versus* further oligomerization a concentration range exists where almost exclusively the dimer species is present (>99%). This allowed us to fully evaluate and quantify the two processes, dimerization and higher aggregate formation, separately. The spectroscopic data for the formation of the higher aggregate was best described by a pentamer fit (5D ⇌ H) and a cooperativity value *σ* of up to 6700 at 298 K was determined.

NOE NMR spectroscopy provided precise insight into the molecular arrangement of the antiparallel aligned chromophores within the dimer aggregate. Regarding the structure of the higher aggregate, its rather small size of six to ten chromophores was consistently indicated by AFM, VPO and DOSY measurements. We propose a *P*-helical stack, build from discrete dimer units based on 1D and 2D NMR experiments as well as the strong CD signal of the higher aggregate, which could be nicely reproduced by TDDFT calculations. The increasing sterical demand of the solubilizing trialkoxyphenyl substituents limit the growth of the stack size, as also observed in other literature examples.^19,20,24–27^ An aliphatic shell can be formed around the polar chromophore core by the dodecyl chains, which would also explain the exceptionally high solubility of the compound even in MCH.

Based on our careful studies of the solvent-, concentration-, temperature-, and time-dependence of the aggregation process of dipolar merocyanine **1** we were able to investigate the fluorescence properties of the individual aggregate species. Accordingly, almost non-emissive merocyanine dyes show aggregation-induced emission enhancement (AIEE) behaviour upon dimerization and the fluorescence is further increased upon growth into larger aggregates. This AIEE behaviour is rationalized by the tight π–π-stacking of the merocyanine dyes in antiparallel dimeric units and further rigidification within more extended oligomeric π-stacks. Increased fluorescence lifetimes and larger Stokes shifts corroborate that the emission can be classified as excimer-type. Thus, this well-characterized two-step dye aggregate system provided valuable insights into the emergence of fluorescence by decrease of the non-radiative decay rates upon aggregation. The system additionally exhibited enhanced CPL for the higher aggregate compared to the dimer species, with a large *g*_lum_ value as high as 0.011.

## Data availability

All supporting data is provided in the ESI.†

## Author contributions

Yvonne Vonhausen: investigation, methodology, visualization, writing. Andreas Lohr: investigation, methodology. Matthias Stolte: methodology, writing, supervision. Frank Würthner: conceptualization, writing, supervision, funding acquisition.

## Conflicts of interest

There are no conflicts to declare.

## Supplementary Material

SC-012-D1SC03813C-s001
