# Supplementary material for: Two-step anti-cooperative self-assembly process into defined π-stacked dye oligomers: insights into aggregation-induced enhanced emission
Source: Chem Sci. 2021 Aug 17;12(37):12302–14. doi: 10.1039/d1sc03813c (PMC8480337; doi:10.1039/d1sc03813c)
Supplement: SC-012-D1SC03813C-s001 [file SC-012-D1SC03813C-s001.pdf]

Electronic Supplementary Material (ESI) for Chemical Science.
This journal is © The Royal Society of Chemistry 2021

Electronic Supplementary Information

Two-step Anti-cooperative Self-assembly Process into Defined π-Stacked Dye Oligomers: Insights into Aggregation-induced Enhanced Emission

Yvonne Vonhausen, Andreas Lohr, Matthias Stolte, Frank Würthner*

Universität Würzburg, Institut für Organische Chemie as well as Center for Nanosystems Chemistry (CNC) and Bavarian Polymer Institute (BPI), Am Hubland, 97074 Würzburg, Germany

*E-mail: wuerthner@uni-wuerzburg.de

Table of Contents

1 Materials and Methods S3

2 Synthesis and Characterization S8

3 Silanization Procedure of Glassware S10

4 UV/Vis Spectroscopy S10

4.1 Solvatochromism S10

4.2 Transition Dipole Moments S11

4.3 Time-dependent UV/Vis Measurements S11

4.4 UV/Vis Absorption of Higher Aggregate at Elevated Temperatures S12

4.5 Higher Aggregate in Cyclohexane S12

4.6 Global Fit Analysis S13

4.7 Multiple Linear Regression (MLR) Analysis S18

4.8 Degree of Aggregated π-faces S19

5 Vapor Pressure Osmometry S20

6 NMR-Experiments S23

6.1 Characterization S23

6.2 Proton Assignment and Structure Elucidation S25

7 CD Spectroscopy S31

8 Fluorescence Spectroscopy S32

8.1 Lifetime Measurements S32

8.2 Relative Determination of the Higher Aggregate Fluorescence Quantum Yield S33

8.3 Solid State Fluorescence S34

9 Higher Aggregate Structure S35

10 Supporting References S36

1 Materials and Methods

Chemicals

Solvents and chemicals were obtained from commercial suppliers and used without further purification, unless otherwise stated. The solvents for UV/Vis absorption, circular dichroism (CD), fluorescence spectroscopy, circular polarized luminescence (CPL), vapor pressure osmometry (VPO) measurements as well as atomic force microscopy (AFM) were of spectroscopic grade and used as received. 1,2,3-Tris(dodecyloxy)benzene **4** was synthesised according to literature known procedure.[S1] Chiral amine **2**[S2] was a gracious donation by Prof. Dr. Klaus Ditrich from the BASF SE in Ludwigshafen, Germany, and had an enantiomeric excess of 99.3%.

Chromatography

Column chromatography was performed on silica gel (Merck Silica 60, particle size 0.04 – 0.063 mm).

Melting Points

Melting points were determined on a Linkam TP 94 heating stage and are uncorrected.

Elemental Analysis

Elemental analyses were conducted on a CHNS 932 analyser (Leco Instruments GmbH)

NMR-Spectroscopy

All spectra were recorded in deuterated solvents at a Bruker Avance III HD 400 or Bruker Avance III HD 600 spectrometer (Germany) using either a 5 mm BBFO probe or a 5 mm DCH cryo-probe, both equipped with z-gradient and a temperature control unit. Chemical shifts are reported in ppm relative to residual solvent signal. For multiplicities, abbreviations are used as follows: s = singlet, d = doublet, t = triplet, m = multiplet, br = broad.

The nuclear Overhauser effect spectrum (^{1}H ^{1}H NOESY) of the dimer of **1** was recorded using the noesygpphpp sequence with a mixing time (d8) of 600 ms. The rotating frame Overhauser effect spectrum (^{1}H ^{1}H ROESY) of the monomer of **1** was recorded using the roesyphpr.2 sequence with a mixing time (p15) of 300 ms.

Diffusion ordered spectroscopy (DOSY) experiments were recorded on a Bruker Avance III HD 600 with a BBFO probe with z gradient and a maximum gradient strength of 50 G cm^{-1}. The DOSY spectra were acquired using the dstebpgp3s (without rotation) or ledbpgp2s (with rotation) pulse sequence. For all experiments, the diffusion gradients were linearly incremented in 32 steps from 2 to 98%. The diffusion time (d20) was set to 50 ms. In case of the dimer of **1** the dstebpgp3s spectrum was evaluated which includes convention compensation. For the higher aggregate no difference was observed between the sequences so the ledbpgp2s spectrum was used for evaluation. Diffusion coefficients were determined by monoexponential fit of the attenuation curves for several peak integrals and averaging of the values. To calculate the hydrodynamic radius according to the Stokes-Einstein equation the value for the dynamic viscosity of undeuterated MCH at the respective temperature was used[S3]. Data processing was performed with the TopSpin 3.5 pl 7 software.

Mass Spectrometry

High-resolution ESI-TOF mass spectrometry for the characterization of compounds **3** and **1** was performed on a microTOF focus instrument (Bruker Daltonics) in positive mode with methanol (MeOH) or acetonitrile (MeCN) as solvent.

UV/Vis Spectroscopy

UV/Vis absorption spectra were recorded on a JASCO V-670 or V-770 spectrometer with a spectral bandwidth of 2 nm and a scan rate of 200 nm/min. The temperature was controlled either by a Peltier element (JASCO) or with a NCP-706 thermostat. Quartz cells (Hellma Analytics) with the following path length were used: 0.01 mm ($c_0 = 1.0 \times 10^{-2}$ to 2.0×10^{-3} M), 0.1 mm ($c_0 = 1.0 \times 10^{-3}$ to 1.7×10^{-4} M), 2 mm ($c_0 = 1.0 \times 10^{-4}$ to 1.7×10^{-5} M), 10 mm ($c_0 = 1.2 \times 10^{-5}$ to 2.2×10^{-6} M), 50 mm ($c_0 = 1.2 \times 10^{-6}$ to 5.2×10^{-7} M), 100 mm ($c_0 = 4.0 \times 10^{-7}$ to 9.8×10^{-8} M). Samples in MCH were prepared in silanized vials and measured in silanized cuvettes (except for cuvettes with a path length of $d < 2$ mm). For concentration-dependent studies, stock solutions were subsequently diluted to adjust the desired concentration. Solutions in MCH were allowed to equilibrate at rt overnight after dilution and 5 to 30 min after a temperature change, before starting the measurement. Repeated measurements were performed to check whether equilibration was complete. Apparent extinction coefficients were calculated according to the Lambert-Beer law and are density corrected for the respective temperatures.

CD Spectroscopy

CD was measured on a JASCO J-810 spectropolarimeter with a spectral bandwidth of 1 nm and a scan rate of 200 nm/min, using the same solvents and quartz cells as for the UV/Vis measurements.

Fluorescence Spectroscopy

Fluorescence spectroscopy was carried out with an FLS-980-D2D2-ST spectrometer (Edinburgh Instruments Ltd., UK). Spectra were corrected against the photomultiplier sensitivity and the lamp intensity. Conventional fluorescence quartz cells (Hellma Analytics) with 10 mm path length were used for standard measurements of solutions. To measure concentrated solutions ($c_0 > 1.0 \times 10^{-4}$ M) a cylindrical cuvette (Hellma Analytics) with a path length of 0.1 mm was used in front-face setup (22.5°). All sample were measured with excitation polarizer 0° and emission polarizer 54.7° (magic angle). The spectral bandwidth was increased until sufficient signal to noise ratio was obtained (5 – 12 nm). To determine relative fluorescence quantum yields the average value at four different excitation wavelengths was used. For the dimer of merocyanine **1** in MCH (λ_{ex} = 485, 490, 495 and 500 nm) *N,N'*-Bis(2,6-diisopropylphenyl)perylene-3,4:9,10-bis(dicarboximide) in $CHCl_3$ was used as a reference (Φ_{Fl} = 100%)[S4]. For the monomer of **1** in CH_2Cl_2 (λ_{ex} = 515, 520, 525 and 530 nm) *N,N'*-Bis(2,6-diisopropylphenyl)-1,6,7,12-tetraphenoxyperylene-3,4:9,10-bis(dicarboximide) in $CHCl_3$ was used as a reference (Φ_{Fl} = 96%)[S4]. All fluorescence lifetimes were determined with an EPL picosecond pulsed diode laser (λ_{ex} = 505.8 nm) for time-correlated single photon counting (TCSPC) with an Edinburgh Instruments FLS980-D2D2-ST spectrometer under magic angle conditions (54.7°). To obtain emission and excitation spectra of merocyanine **1** in the solid state, the solid sample was manually applied on a quartz surface and measured in front-face setup with the FLS-980-D2D2-ST spectrometer (Edinburgh Instruments Ltd., UK). Absolute quantum yields of the solid material were determined on a Hamamatsu Absolute PL Quantum Yield Measurement System *CC9920-02*.

CPL Spectroscopy

CPL spectra were recorded with a customised JASCO CPL-300/J-1500 hybrid spectrometer. The dimer sample was measured in a conventional fluorescence quartz cell (Hellma Analytics) with 10 mm path length. The excitation and emission bandwidth were set to 36 nm and 11 nm,

respectively. The HAT photomultiplier current was 995 V and the spectrum was recorded with a scan speed of 50 nm/min and a D.I.T. of 2 sec. 30 accumulations were measured.
For the higher aggregate sample in MCH a cylindrical cuvette (Hellma Analytics) with a path length of 1 mm was used. The excitation and emission bandwidth were set to 36 nm and 10 nm, respectively. The HAT photomultiplier current was 950 V and the spectrum was recorded with a scan speed of 50 nm/min and a D.I.T. of 2 sec. 3 accumulations were measured.

Atomic Force Microscopy

AFM measurements were performed under ambient conditions using a Bruker Multimode 8 SPM system operating in tapping mode in air. Silica cantilevers (OMCL-AC200TS, Olympus) with a resonance frequency of ~150 kHz and a spring constant of ~10 N m^{-1} were used. The solution of the sample in MCH was spin-coated onto *n*-tetradecylphosphonic acid(TPA)-modified Si/SiO_2 (100 nm)/AlO_x (8 nm) substrates.

VPO Measurement

The vapor pressure osmometry measurements were performed on a KNAUER osmometer with a universal temperature measurement unit. For the measurements in MCH the measurement chamber was heated to 318 K and the chamber lid with the syringes to 320 K. Benzil (M = 210.23 g/mol) was purchased from Merck KGaA, Darmstadt Germany. The polystyrene standard PS5270 was purchased from PSS – Polymer Standards Service, Mainz, Germany. The number average molar mass is given as M_n = 5270 g/mol. Samples in MCH were prepared by diluting the most concentrated solution and allowed to equilibrate overnight. The measurement value (*MV*) of a sample was determined as the average of three to five measurements.

Computational Details

Geometry-optimization for monomer (M) and dimer (D) of merocyanine **1** was performed at the density functional theory (DFT) level using the B97D3 functional[S5] including dispersion correction and the def2-SVP basis[S6] as implemented in the Gaussian 16 program package[S7]. The polarizable continuum model (PCM)[S8-10] was used with MCH as solvent. Dodecyl chains were replaced by methyl groups to reduce computational effort. Frequency calculations were performed to verify the optimized geometries as stationary minima. The rotational barrier around the C–N bond of the tetralin substituent of the monomer in the gas phase was calculated by performing a relaxed potential energy surface scan for a 180° rotation around this bond in

5° steps and calculating the energy difference between the highest and lowest energy structure. For this the B97D3 functional[S5] and the def2-SVP basis set[S6] was used.

Due to the large size of the proposed decamer stack (H), the geometry was optimized using the semi-empirical PM7 Hamiltonian[S11] within the MOPAC software package[S12], which also accounts for dispersion interaction. Dodecyl chains were replaced by methyl groups to reduce computational effort. Frequency calculations were performed to verify the optimized geometry as a stationary minimum. TDDFT calculations were performed on the geometry-optimized decamer stack (the benzene substituents were replaced by methyl groups after structure optimization to reduce computational effort for TDDFT calculations) using the ωB97[S13] functional and the def2SVP basis set[S6] as implemented in the Gaussian 16 program package[S7]. The polarizable continuum model (PCM)[S8-10] was used with MCH as solvent. The oscillator strength for the first 15 states were calculated and the CD spectrum was simulated with the help of GaussView 5[S14]. The half with at half hight was set to 0.18 eV and the spectrum was shifted 0.64 eV to lower energies to fit the experimental maximum.

2 Synthesis and Characterization

Hydroxypyridone 3

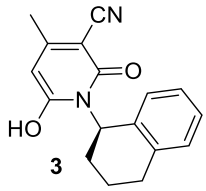

3

Ethyl cyanoacetate (1.15 g, 10.2 mmol) and chiral amine **2** (1.47 g, 10.0 mmol) were refluxed in MeOH (10 mL) for 48 h. The solvent was evaporated under reduced pressure. Ethyl acetoacetate (1.27 mL, $\rho = 1.06$ g/mL, 10.0 mmol) and piperidine (1 mL, $\rho = 0.86$ g/mL, 10.1 mmol) were added, and the mixture was stirred at 100 °C for 24 h. The solvent was removed under reduced pressure and the residue dissolved in MeOH. The pH value was adjusted to 1 with 32% aqueous HCl. After precipitation at room temperature, the product was filtered off, washed with distilled water and diethylether and dried *in vacuo*. Recrystallization from an ethyl acetate ethanol mixture gave the pure pyridone **3**.

Yield: 580 mg (2.07 mmol, 21%).

Mp: 250–252 °C.

^{1}H NMR (400 MHz, DMSO-d_6, 295 K): $\delta = 7.08$ (m, 3H), 6.65 (d, $J = 6.7$ Hz, 1H), 6.36 (br, 1H), 5.96 (br, 1H), 5.48 (br, 1H), 2.76 (m, 2H), 2.33 (m, 1H), 2.21 (s, 3H), 2.00 (m, 2H), 1.76 (m, 1H) ppm.

^{13}C NMR (101 MHz, DMSO-d_6, 295 K): $\delta = 161.5$, 160.7, 158.5, 137.0, 136.2, 128.8, 126.1, 126.0, 124.2, 117.8, 93.5, 88.0, 51.0, 29.0, 26.6, 22.6, 20.5 ppm.

HRMS (ESI, pos. mode, MeOH): *m*/*z* calcd. for $C_{17}H_{16}N_2O_2Na$ ([M+Na]$^+$) 303.11040, found 303.11039.

Anal. calcd. for $C_{17}H_{16}N_2O_2$ (280.33): C, 72.84; H, 5.75; N, 9.99. Found: C, 72.70; H, 5.77; N, 10.02.

Merocyanine 1

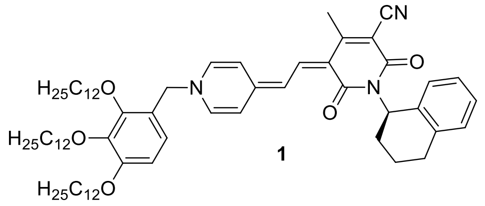

Pyridone **3** (196 mg, 699 μmol) and *N,N′*-diphenylformamidine (137 mg, 698 μmol) were stirred in Ac_2O (1.20 mL) at room temperature for 15 min. The mixture was then heated to 90 °C for additional 30 min to complete the reaction. After the reaction mixture cooled to room temperature, pyridinium salt **5** (520 mg, 636 μmol) and KOAc (98.1 mg, 1.00 mmol) were added, and the mixture was heated at 100 °C for 14 h. The solution was concentrated *in vacuo* and the crude product purified by column chromatography on silica using dichloromethane/methanol (98.5:1.5) as eluent. After evaporation of the solvent, the compound was again dissolved in pure dichloromethane, filtrated, concentrated and dried in high vacuum (2×10^{-3} mbar) at 40 °C for 3 h.

Yield: 124 mg (121 μmol, 19%).

Mp: 70 °C.

^{1}H NMR (400 MHz, CD_2Cl_2, 295 K): δ = 7.78 (br, 2H), 7.74–7.50 (br, 2H), 7.32 (br, 2H), 7.08-6.95 (m, 4H), 6.83 (d, J = 7.6 Hz, 1H), 6.66 (d, J = 8.6 Hz, 1H), 6.27 (br, 1H), 5.12 (s, 2H), 4.03 (t, J = 7.0 Hz, 2H), 3.96 (t, J = 6.4 Hz, 2H), 3.92 (t, J = 6.6 Hz, 2H), 2.97 (m, 1H), 2.75 (d, J = 15.9 Hz, 1H), 2.54 (m, 1H), 2.42 (s, 3H), 2.06 (m, 1H), 1.93 (m, 1H), 1.87 – 1.77 (m, 3H), 1.75-1.60 (m, 4H), 1.53–1.21 (m, 54H), 0.88 (t, J = 6.9 Hz, 9H) ppm.

^{13}C NMR (151 MHz, CD_2Cl_2, 295 K): δ = 164.3, 163.3, 157.5, 156.6, 156.1, 151.9, 142.0, 140.6, 140.0, 138.8, 137.6, 129.0, 126.0, 125.5, 125.4, 125.0, 120.2, 119.7, 118.3, 112.9, 108.3, 107.6, 74.3, 74.0, 69.3, 58.8, 50.6, 32.3, 30.9, 30.7, 30.2, 30.1 (three peaks), 30.0, 29.9, 29.8, 29.7, 27.0, 26.5 (two peaks), 26.4, 23.7, 23.1, 18.9, 14.3 ppm.

HRMS (ESI, pos. Mode, MeCN): *m/z* calcd for $C_{67}H_{100}N_3O_5$ ($[M+H]^+$) 1026.7657, found 1026.7646.

Anal. calcd. for $C_{67}H_{99}N_3O_5$ (1026.52): C, 78.39; H, 9.72; N, 4.09. Found: C, 78.20; H, 9.91; N, 4.38.

UV/Vis (CH_2Cl_2, 298 K, $c_0 = 6 \times 10^{-6}$ M): λ_{max} (ε) = 549 nm (116000 $M^{-1}cm^{-1}$).

3 Silanization Procedure of Glassware

For the silanization of one commercial 1 × 1 cm cuvette a mixture of freshly distilled trimethylsilylchloride (4.60 mL, 50.0 mmol) and NaI (7.50 g, 50.0 mmol) in dry acetonitrile (200 mL) was placed in a 250 mL round bottom flask under nitrogen atmosphere. The cuvette was immersed and the mixture heated to 90 °C overnight. After cooling, the cuvette was washed with distilled water, acetone and CH_2Cl_2. The amount of silanization reagents was adapted for other glassware by taking into account the approximate outer and inner surface of the respective cuvettes or glass vials as well as the inner surface of the reaction flask.

4 UV/Vis Spectroscopy

4.1 Solvatochromism

When comparing the absorption spectra of the monomer of merocyanine **1** in different solvents with increasing polarity (Fig. S1), a pronounced negative solvatochromism can be observed. This is in accordance with the decrease in dipole moment upon optical excitation reported in literature for this kind of dipolar chromophore (μ_g = 17.1 D, μ_e = 12.6 D)[S15]. Also for the dimer a negative but smaller solvatochromic effect can be observed.

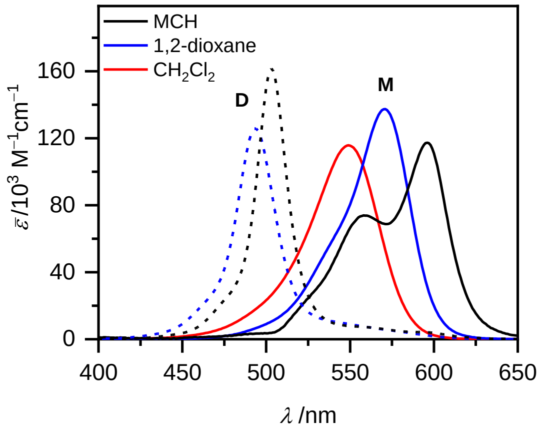

Fig. S1 UV/Vis absorption spectra of merocyanine **1** as monomer (M, solid lines) and dimer (D, dashed lines) in dichloromethane (CH_2Cl_2, red, $c_0 = 1.3 \times 10^{-3}$ M), 1,4-dioxane (blue, global fit analysis) and MCH (MCH, black, global fit analysis) at 298 K.

4.2 Transition Dipole Moments

The electronic transition dipole moments (μ_{eg}) were calculated according to equation (S1). I_A is the integrated absorption, N_A the Avogadro constant, ε_0 the vacuum permittivity, c_0 the speed of light in vacuum and h the Planck constant.

$$|\mu_{eg}^2| = \frac{3\,I_A}{S}\,, \tag{S1}$$

With S defined as:

$$S = \frac{2\pi^2 N_A}{\varepsilon_0 c_0 h \ln 10} = 2.9356\ \times 10^{60}\ \frac{1}{\mathrm{mol\ C^2}}\,. \tag{S2}$$

4.3 Time-dependent UV/Vis Measurements

The higher aggregate shows kinetic stability in MCH upon dilution from $c_0 = 1.1 \times 10^{-3}$ M to $c_0 = 1.1 \times 10^{-5}$ M at 298 K. Time-dependent UV/Vis measurements show the slow transition from the higher aggregates to the dimers (Fig. S2).

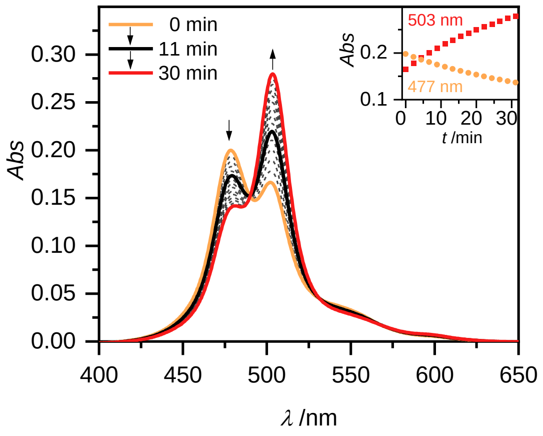

Fig. S2 Time-dependent UV/Vis absorption spectra of a sample of merocyanine **1** in MCH 0 – 30 min after dilution form 1.1×10^{-3} M to 1.1×10^{-5} M at 298 K. Arrows indicate the spectral changes over time.

After rapid cooling from 353 to 298 K, two processes can be observed in the UV/Vis spectra of a concentrated solution of merocyanine **1** in MCH ($c_0 = 8.9 \times 10^{-4}$ M): 1) The dimer species that is formed at 353 K reassembles and 2) the absorption band of higher aggregate slowly returns from a more broad shape to its original room temperature shape of an H-aggregate with a maximum at $\lambda = 477$ nm and a shoulder at $\lambda \sim 540$ nm (Fig. S3).

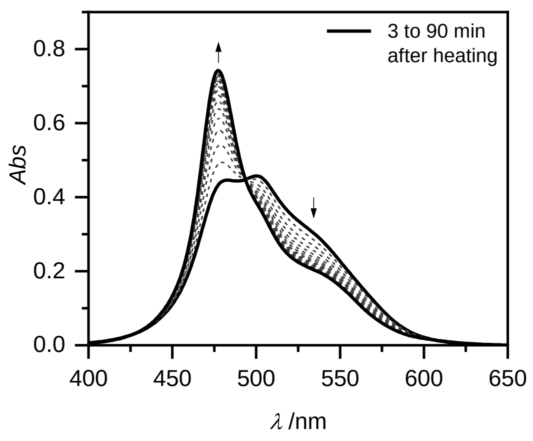

Fig. S3 Time-dependent UV/Vis absorption spectra of a sample of merocyanine **1** in MCH (c_0 = 8.9 × 10^{-4} M) heated up to 353 K for 10 min and then measured at 298 K after rapid cooling. Arrows indicate the spectral changes over time.

4.4 UV/Vis Absorption of Higher Aggregate at Elevated Temperatures

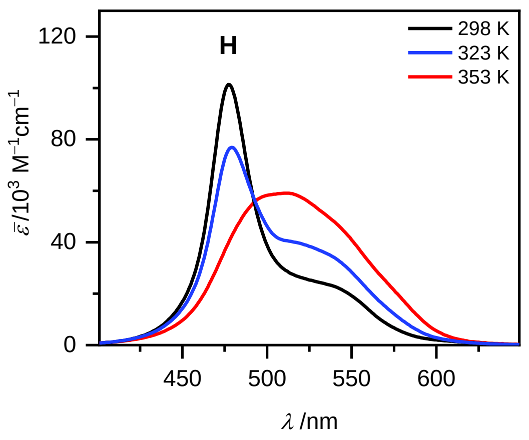

Fig. S4 Calculated UV/Vis absorption spectra of the higher aggregate of merocyanine **1** from global fit analysis of the concentration-dependent spectra in MCH at 298 K (black), 323 K (blue) and 353 K (red) according to the pentamer model.

4.5 Higher Aggregate in Cyclohexane

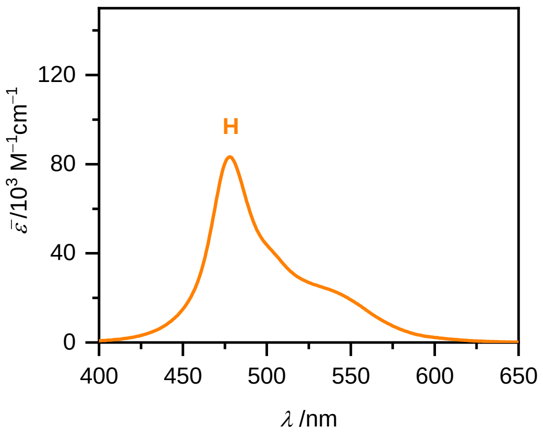

Fig. S5 UV/Vis absorption spectrum of higher aggregate of **1** in cyclohexane (c_0 = 1 × 10^{-3} M, 298 K).

4.6 Global Fit Analysis

A global fit algorithm as introduced in previous literature examples[S16-18] was used to evaluate the concentration-dependent UV/Vis studies according to different aggregation models. The dimer, trimer, tetramer, pentamer and isodesmic model[S18, 19] were applied.

The dimer fit[S19] (2 M ⇌ D) was applied to selected data from the dilution series at 353 K in MCH, showing the transition from dimer (D) to monomer (M) (Fig. S6a). The experimental apparent extinction data ($\bar{\varepsilon}$) of samples with a total molecular concentration c_0 from several dilution series is compared to the simulated curve for the dimer model according to

$$\bar{\varepsilon} = (\varepsilon_{\mathrm{M}} - \varepsilon_{\mathrm{D}})\frac{\sqrt{8K_{\mathrm{D}}c_0+1}-1}{4K_{\mathrm{D}}c_0} + \varepsilon_{\mathrm{D}}, \quad \text{(S3)}$$

with the extinction of the pure monomer (ε_{M}) and dimer (ε_{D}) species, as well as the dimerization constant $K_{\mathrm{D}} = 4.5 \times 10^6\ \mathrm{M}^{-1}$ obtained from global fit analysis (Fig. S6b,c).

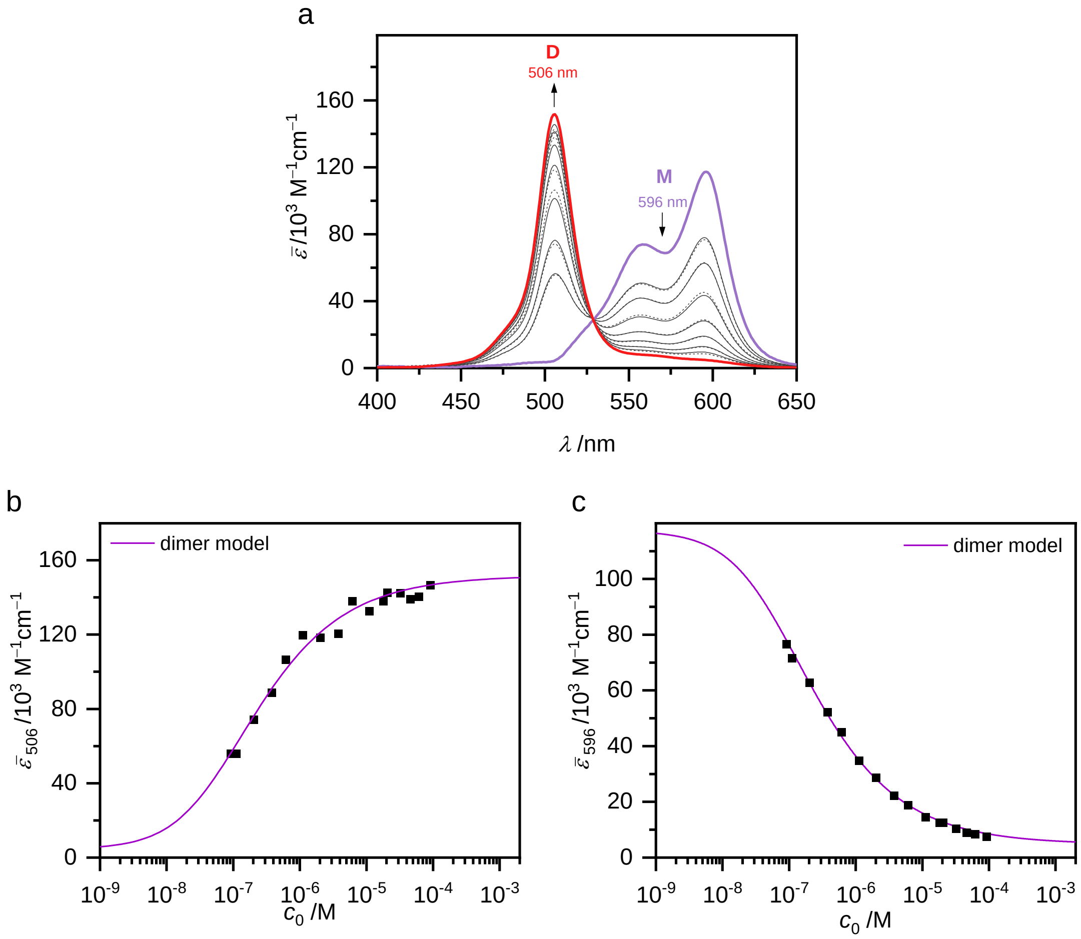

Fig. S6 a) Concentration-dependent UV/Vis absorption spectra (dashed grey lines) of merocyanine **1** in MCH at 353 K in comparison with calculated spectra (solid grey lines) from global fit analysis according to the dimer model. Arrows indicate the spectral changes upon increasing the concentration from $c_0 = 9.1 \times 10^{-8} - 6.1 \times 10^{-5}$ M. Colored spectra are calculated spectra for the individual monomer (M, violet) and dimer (D, red) species. b/c) Concentration-dependent experimental extinction coefficients at 353 K at the maximum of the dimer (b, 506 nm) and monomer (c, 596 nm) absorption band in comparison with the simulated curve according to the dimer model (eq. (S3)).

The trimer, tetramer and pentamer fit[S18] (n D ⇌ H, n = 3,4,5) were applied to selected data of the dilution series at 298 (Fig. S7), 323 (Fig. S8) and 353 K (Fig. S9) in MCH, showing the transition from the higher aggregate (H) to the dimer (D). The experimental apparent extinction data ($\bar{\varepsilon}$) of samples with a total molecular concentration c_0 from several dilution series is compared to the simulated curves for the individual models, with the extinction coefficients of the pure dimer (ε_D) and higher aggregate (ε_H) species, as well as the binding constant per binding side K_n obtained from global fit analysis. The formation of defined aggregates out of n dimers is described by expression (S4). In combination with Lambert-Beer's law (S5) K_n can be expressed as equation (S6).

$$c_0 = 2\,c_\mathrm{D} + 2\mathrm{n}\,c_\mathrm{H}, \tag{S4}$$

$$c_0 \cdot \bar{\varepsilon} = 2\,c_\mathrm{D} \cdot \varepsilon_\mathrm{D} + 2\mathrm{n}\,c_\mathrm{H} \cdot \varepsilon_\mathrm{H}, \tag{S5}$$

$$K_\mathrm{n} = \sqrt[\mathrm{n}-1]{\frac{c_\mathrm{H}}{c_\mathrm{D}{}^\mathrm{n}}} = \sqrt[\mathrm{n}-1]{\frac{\left(c_0 - \frac{c_0(\bar{\varepsilon}-\varepsilon_\mathrm{H})}{(\varepsilon_\mathrm{D}-\varepsilon_\mathrm{H})}\right)\cdot\frac{1}{2\mathrm{n}}}{\left(\frac{c_0(\bar{\varepsilon}-\varepsilon_\mathrm{H})}{2(\varepsilon_\mathrm{D}-\varepsilon_\mathrm{H})}\right)^\mathrm{n}}}. \tag{S6}$$

Additionally, the isodesmic fit[S19] was applied to selected data of the dilution series at 298 (Fig. S7), 323 (Fig. S8) and 353 K (Fig. S9) in MCH, showing the transition from higher aggregate (H) to dimer (D) (n D ⇌ H). The experimental apparent extinction data ($\bar{\varepsilon}$) of samples with a total molecular concentration c_0 from several dilution series is compared to the simulated curve for the isodesmic model, with the extinction coefficients of the pure dimer (ε_D) and higher aggregate (ε_H) species, as well as the binding constant K_n obtained from global fit analysis. For the simulated curves according to the isodesmic aggregation of *n* dimers, the following equation applies:

$$\bar{\varepsilon} = (\varepsilon_\mathrm{D} - \varepsilon_\mathrm{H})\frac{2K_\mathrm{n}c_0 + 1 - \sqrt{4K_\mathrm{n}c_0+1}}{2K_\mathrm{n}{}^2 c_0{}^2} + \varepsilon_\mathrm{H}. \tag{S7}$$

The binding constants for the formation of the higher aggregate out of dimers (*n* D ⇌ H) at different temperatures, obtained from global fit analysis according to the different aggregation models, are summarized in Table S1.

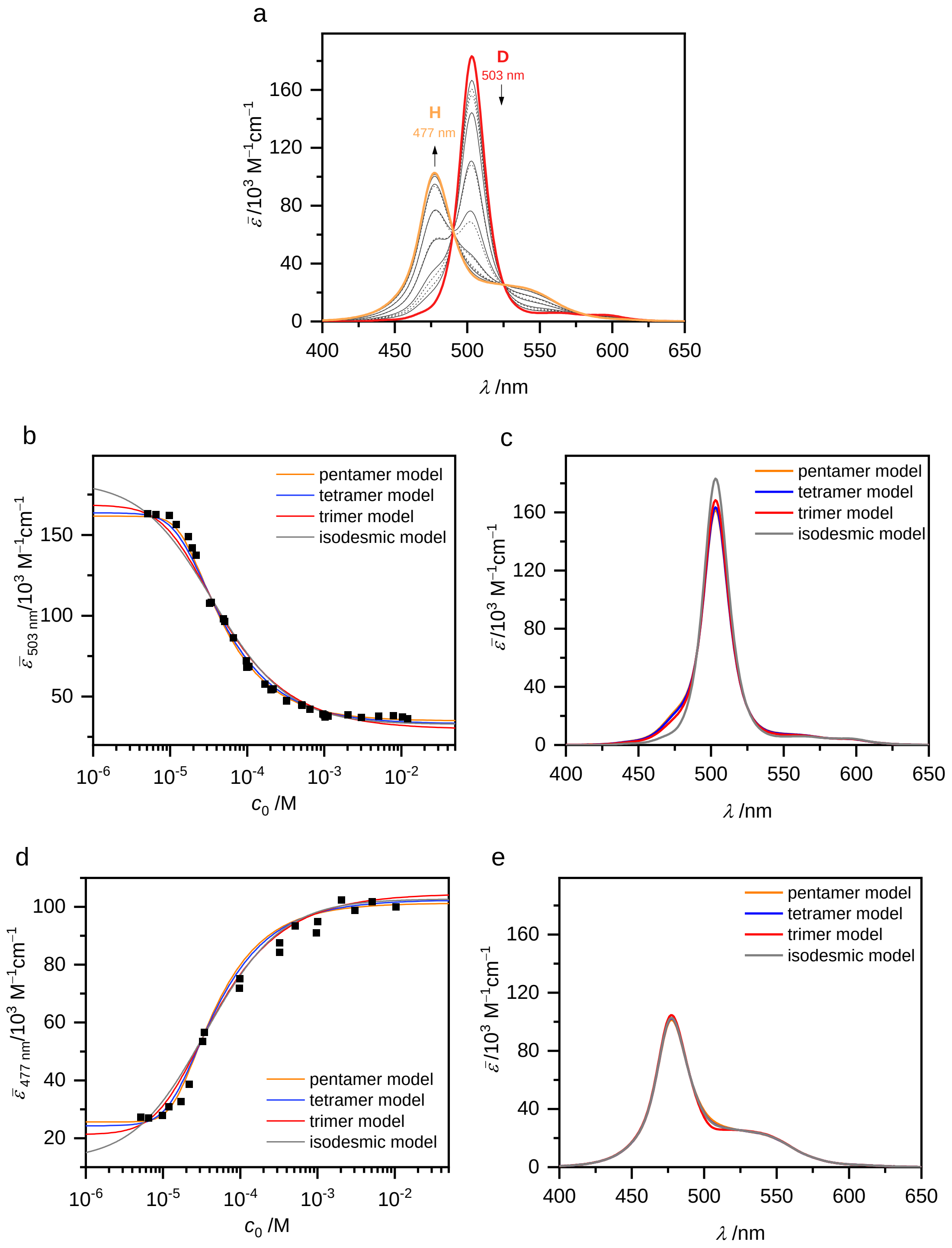

Fig. S7 a) Concentration-dependent UV/Vis absorption spectra (dashed grey lines) of merocyanine **1** in MCH at 298 K in comparison with calculated spectra (solid grey lines) from global fit analysis according to the pentamer model. Arrows indicate the spectral changes upon increasing the concentration from $c_0 = 4.0 \times 10^{-6} - 5.1 \times 10^{-3}$ M. Colored spectra are calculated spectra for the individual dimer (D, red) and higher aggregate (H, orange) species. b, d) Concentration-dependent experimental extinction coefficients of several independent dilution experiments at 298 K at the maximum of the dimer (b, 503 nm) or higher aggregate (d, 477 nm) absorption band in comparison with simulated curves according to different aggregation models (eq. (S6) and (S7)). c, e) Comparison of calculated spectra of dimer (c) and higher aggregate (e) from global fit analysis according to different models.

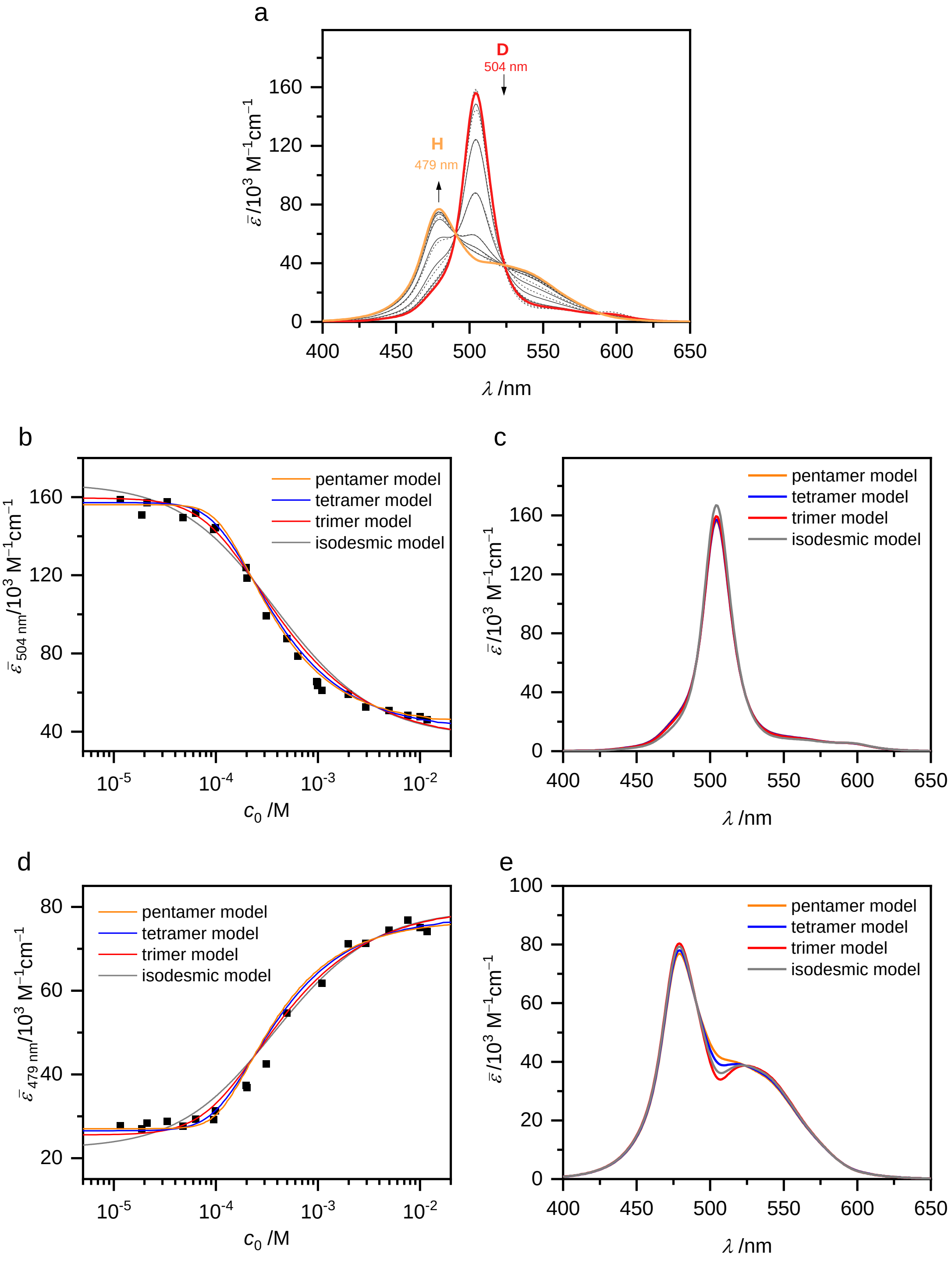

Fig. S8 a) Concentration-dependent UV/Vis absorption spectra (dashed grey lines) of merocyanine **1** in MCH at 323 K in comparison with calculated spectra (solid grey lines) from global fit analysis according to the pentamer model. Arrows indicate the spectral changes upon increasing the concentration from $c_0 = 1.2 \times 10^{-5} - 1.0 \times 10^{-2}$ M. Colored spectra are calculated spectra for the individual dimer (D, red) and higher aggregate (H, orange) species. b, d) Concentration-dependent experimental extinction coefficients of several independent dilution experiments at 323 K at the maximum of the dimer (b, 504 nm) or higher aggregate (d, 479 nm) absorption band in comparison with simulated curves according to different aggregation models (eq. (S6) and (S7)). c, e) Comparison of calculated spectra of dimer (c) and higher aggregate (e) from global fit analysis according to different models.

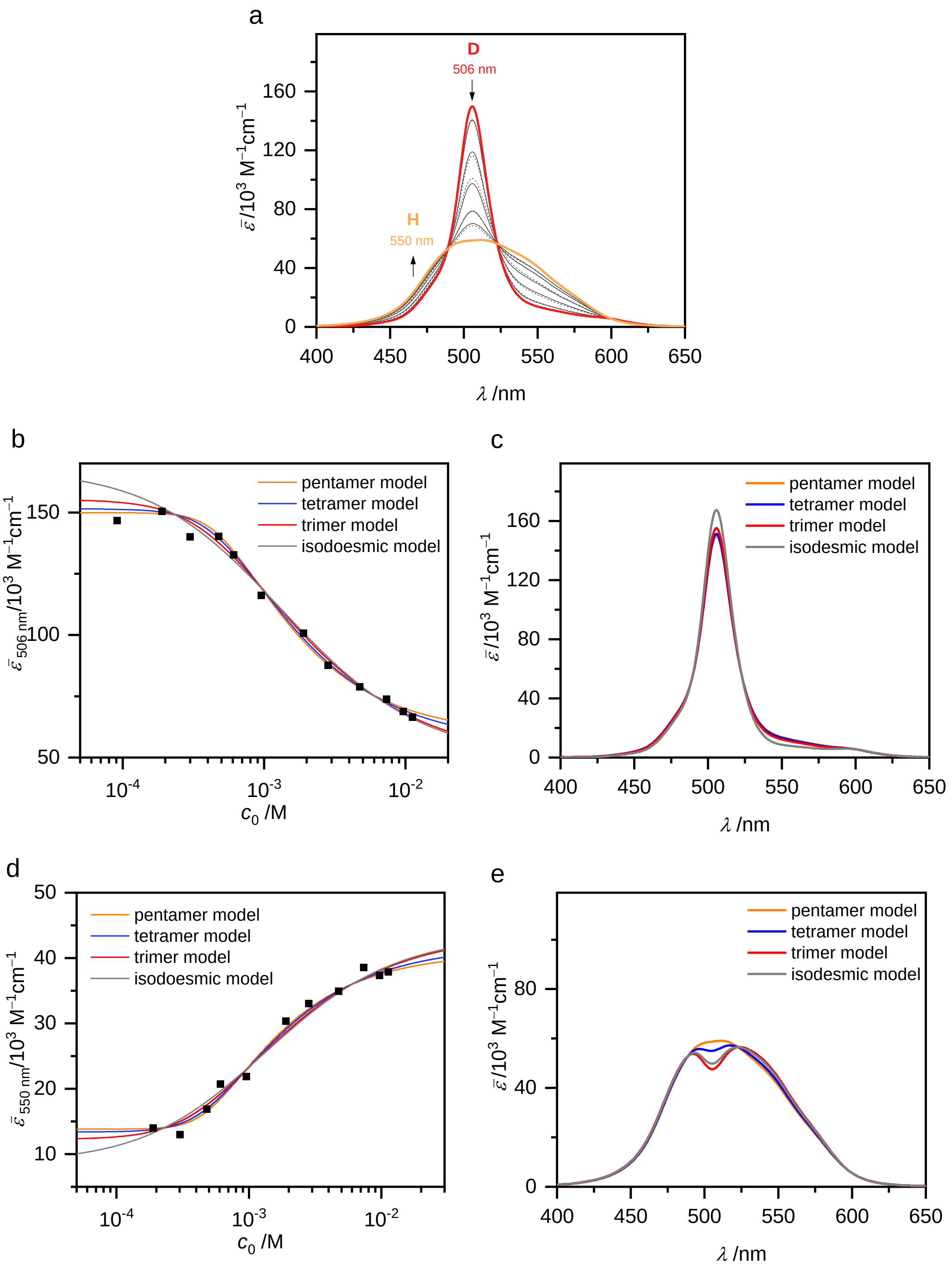

Fig. S9 a) Concentration-dependent UV/Vis absorption spectra (dashed grey lines) of merocyanine **1** in MCH at 353 K in comparison with calculated spectra (solid grey lines) from global fit analysis according to the pentamer model. Arrows indicate the spectral changes upon increasing the concentration from $c_0 = 1.9 \times 10^{-4} – 9.7 \times 10^{-3}$ M. Colored spectra are calculated spectra for the individual dimer (D, red) and higher aggregate (H, orange) species. b, d) Concentration-dependent experimental extinction coefficients of several independent dilution experiments at 353 K at the maximum of the dimer (b, 506 nm) or higher aggregate (d, 550 nm) absorption band in comparison with simulates curves according to different aggregation models (eq. (S6) and (S7)). c, e) Comparison of calculated spectra of dimer (c) and higher aggregate (e) from global fit analysis according to different models.

Table S1 Overview of the binding constants K_n for the formation of the higher aggregate (n D ⇌ H) obtained from global fit analysis of concentration-dependent UV/Vis data at different temperatures according to different aggregation models.

	K_n (298 K) /M^{-1}	K_n (323 K) /M^{-1}	K_n (353 K) /M^{-1}
Pentamer	6.0 × 10^4	7.5 × 10^3	1.8 × 10^3
Tetramer	5.5 × 10^4	6.6 × 10^3	1.6 × 10^3
Trimer	4.9 × 10^4	5.3 × 10^3	1.3 × 10^3
Isodesmic	3.2 × 10^4	3.0 × 10^3	0.8 × 10^3

4.7 Multiple Linear Regression (MLR) Analysis

To determine the concentration of molecules present as monomer (x_M), dimer (x_D) and higher aggregate (x_H) in a sample with a total molecular concentration c_0, multiple linear regression (MLR) analysis was performed. Based on Lambert-Beer's law (equation (S8)) the extinction $E(\lambda)$ can be deconvoluted into the contributions of the individual species ($\varepsilon_M(\lambda)$, $\varepsilon_D(\lambda)$, $\varepsilon_H(\lambda)$) for each cuvette thickness d.

$$E(\lambda) = c_0 \varepsilon(\lambda) d = [x_M\, \varepsilon_M\, (\lambda) + x_D\, \varepsilon_D(\lambda) + x_H\, \varepsilon_H(\lambda)] d. \tag{S8}$$

By MLR analysis the factors x_M, x_D and x_H (which are defined to be ≥ 0) were obtained. Please note, that x_D and x_H are not the actual concentrations of dimer and higher aggregate but the concentration of monomeric molecules present as dimer (D) or higher aggregate (H) species as defined by the way the extinction coefficient is calculated. The spectra calculated according to equation (S8) from the extinction spectra of the individual species and the coefficients obtained by MLR analysis are in excellent agreement with the experimental spectra (Fig. S10). Additionally, the calculated total concentration $c_\Sigma = x_M + x_D + x_H$ matches the experimentally balanced concentration c_0 well (Fig. S10, insets), which confirms the validity of the method.

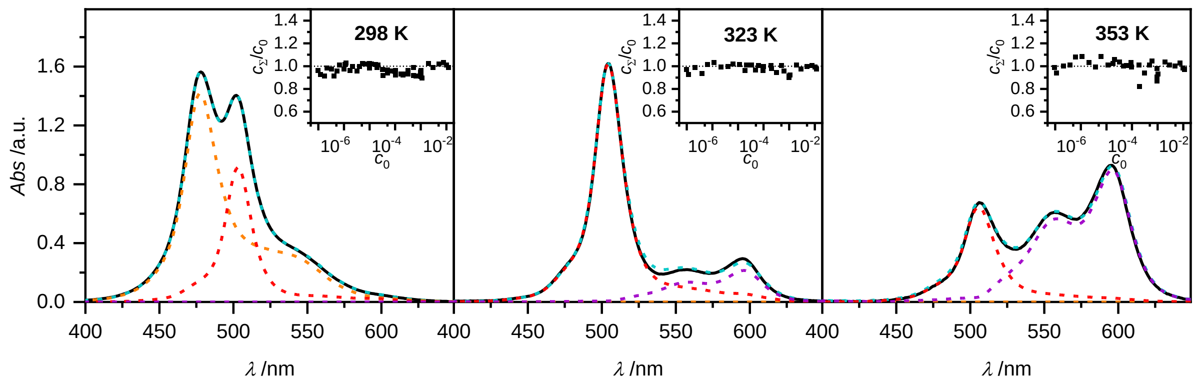

Fig. S10 Experimental UV/Vis absorption spectra (black solid line) of merocyanine **1** and calculated spectra according to equation (S9) (turquoise dashed line) as well as the partial contributions of monomer (violet dashed line), dimer (red dashed line) and higher aggregate (orange dashed line) at $c_0 = 1.0 \times 10^{-4}$ M (298 K), $c_0 = 3.9 \times 10^{-7}$ M (323 K) and $c_0 = 9.8 \times 10^{-8}$ M (353 K). Insets show the deviation of the calculated total concentration $c_\Sigma = x_M + x_D + x_H$ from MLR analysis with respect to the balanced concentration c_0.

4.8 Degree of Aggregated π-faces

In general, the degree of aggregated π-faces $\alpha_{\text{agg-}\pi}$ is defined as the number N of occupied π-faces divided by the total number of π-faces (equation (S9)).

$$\alpha_{\text{agg}-\pi} = \frac{N_{\text{occupied }\pi-\text{faces}}}{N_{\text{total }\pi-\text{faces}}}. \quad \text{(S9)}$$

For the dimerization of two monomers (M) according to 2M ⇌ D applies:

$$c_0 = c_M + 2c_D\,. \quad \text{(S10)}$$

$$c_0 \cdot \bar{\varepsilon} = c_M \cdot \varepsilon_M + 2\,c_D \cdot \varepsilon_D \rightarrow \frac{2\,c_D}{c_0} = \frac{\bar{\varepsilon} - \varepsilon_M}{\varepsilon_D - \varepsilon_M}\,. \quad \text{(S11)}$$

$$\alpha_{\text{agg}-\pi} = \frac{2\,c_D}{2\,c_0} = \left(\frac{\bar{\varepsilon} - \varepsilon_M}{\varepsilon_D - \varepsilon_M}\right) \cdot 0.5. \quad \text{(S12)}$$

For the formation of a higher aggregate (H) out of five dimers (D) according to 5D ⇌ H applies:

$$c_0 = 2c_D + 10c_H. \quad \text{(S13)}$$

$$c_0 \cdot \bar{\varepsilon} = 2\,c_D \cdot \varepsilon_D + 10\,c_H \cdot \varepsilon_H \rightarrow \frac{10\,c_H}{c_0} = \frac{\bar{\varepsilon} - \varepsilon_D}{\varepsilon_H - \varepsilon_D}\,. \quad \text{(S14)}$$

$$\alpha_{\text{agg}-\pi} = \frac{20\,c_H + 2\,c_D}{2\,c_0} = \frac{10\,c_H + c_0}{2\,c_0} = \left(\frac{10\,c_H}{c_0}\right) \cdot 0.5 + \frac{c_0}{2\,c_0} = \left(\frac{\bar{\varepsilon} - \varepsilon_D}{\varepsilon_H - \varepsilon_D}\right) \cdot 0.5 + 0.5. \quad \text{(S15)}$$

For simplification it is assumed, that for H all 20 π-faces are occupied.

5 Vapor Pressure Osmometry

The molecular weight of the higher aggregate of merocyanine **1** ($M_{monomer}$ = 1026.55 g/mol) was determined by vapor pressure osmometry (VPO). This method allows to determine the total osmolality of solutions. Solutions containing solutes have lower vapor pressure than the pure solvent, which leads to a vapor pressure difference, and thus to a temperature difference (ΔT) at the thermistors during the measurement. This ΔT is proportional to the number of particles dissolved in the solution. Benzil (M = 210.23 g/mol) and polystyrene PS5270 (M_n = 5270 g/mol) were used as standards to determine the relation of measurement value (MV) and osmolality for the device and the applied measurement conditions. MCH was used as solvent and the measurements were performed at 318 K to ensure saturation of the gas phase of the cell with solvent vapor.

To evaluate the data two different methods can be applied. The first one assumes a linear relation between MV and the number of particles. A linear fit was applied to the data of the two standards with a fixed intercept at $y = 0$. With the slope s of these fit curves, the concentration c in mol kg^{-1} (moles of particles per kg solution) of unknown samples can be directly determined from the measurement value MV according to equation (S16). In this way, the average number $\langle X_n \rangle$ of molecules incorporated in these particles can be calculated.

$$c\left(\frac{mol}{kg}\right) = \frac{MV}{s\left(\frac{kg}{mol}\right)}\,. \tag{S16}$$

The slope obtained from the linear fit of benzil and PS5270 reference measurements was identical (s = 562 kg mol^{-1}) and $\langle X_n \rangle$ was calculated for samples with different concentrations (in g of compound per kg solution, Fig. S11a).

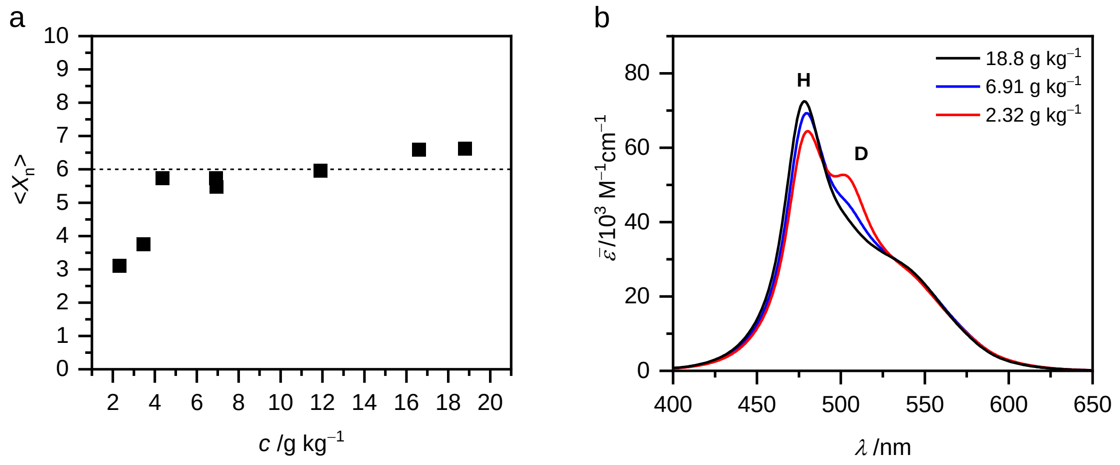

Fig. S11 a) Average number of molecules incorporated into the higher aggregate of merocyanine **1** (MCH, 318 K) calculated from the VPO measurement values (*MV*) of samples of **1** with varying concentrations. A linear relation between *MV* and particle concentration is assumed. b) UV/Vis absorption spectra of samples of merocyanine **1** in MCH at 318 K used for VPO measurements.

The value rises with concentration up to $<X_n>$ = 6.6. This is in accordance with the fact, that in the lower concentrated samples at 318 K mixtures of dimer and higher aggregates are present according to the UV/Vis spectra (Fig. S11b).

The second way to evaluate the data from VPO measurements considers that for large molecules (M > 500 g/mol) the relation between *MV* and the number of particles is not necessarily linear. It is recommended to use a standard with a similar molecular weight as expected for the analyte (here PS5270). The measurement values *MV* divided by the concentration (in mol/kg for the reference and in g/kg for the analyte) are plotted against the concentration (Fig. S12). A linear fit is applied. For the data of merocyanine **1**, the data points for the two lowest concentrations are excluded, since these samples are not fully aggregated under the measurement conditions. K_{cal} and K_{meas} are obtained as the intercept at $c = 0$. The molecular weight of the analyte can then be calculated according to:

$$M = \frac{K_{\mathrm{cal}}}{K_{\mathrm{meas}}}\,. \qquad \text{(S17)}$$

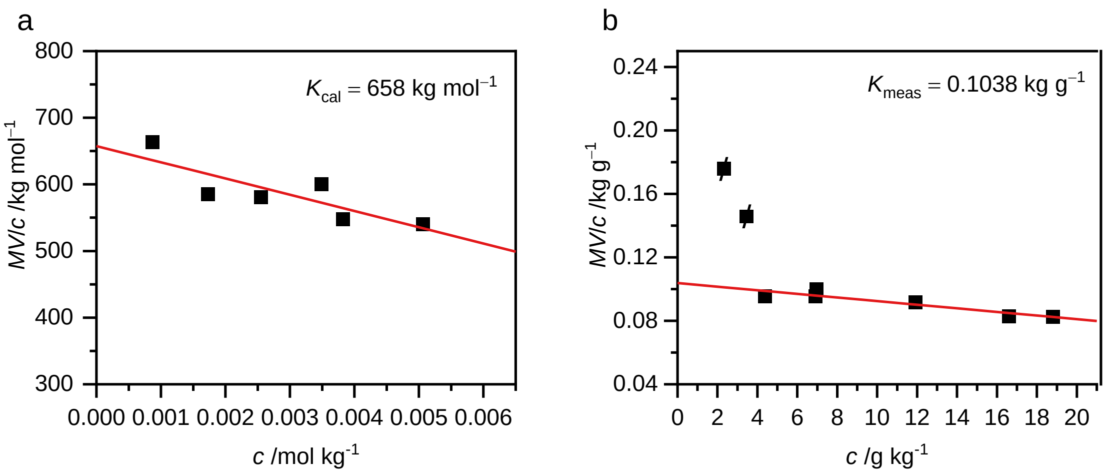

Fig. S12 VPO measurement values (*MV*) divided by the concentration *c* as a function of the sample concentration of the reference PS5270 (a) and merocyanine **1** (b) in MCH at 318 K. The *K* values are the interpolated *y*-values of the linear fit at $c = 0$.

By this, an average weight of the higher aggregate particles of $M = 6339$ g/mol was obtained, which corresponds to $\langle X_n \rangle = 6.2$. Thus, both evaluation methods gave very similar results, indicating an average number of six molecules (trimer of dimers) for the higher aggregate species formed by merocyanine **1** in MCH at 318 K.

6 NMR-Experiments

6.1 Characterization

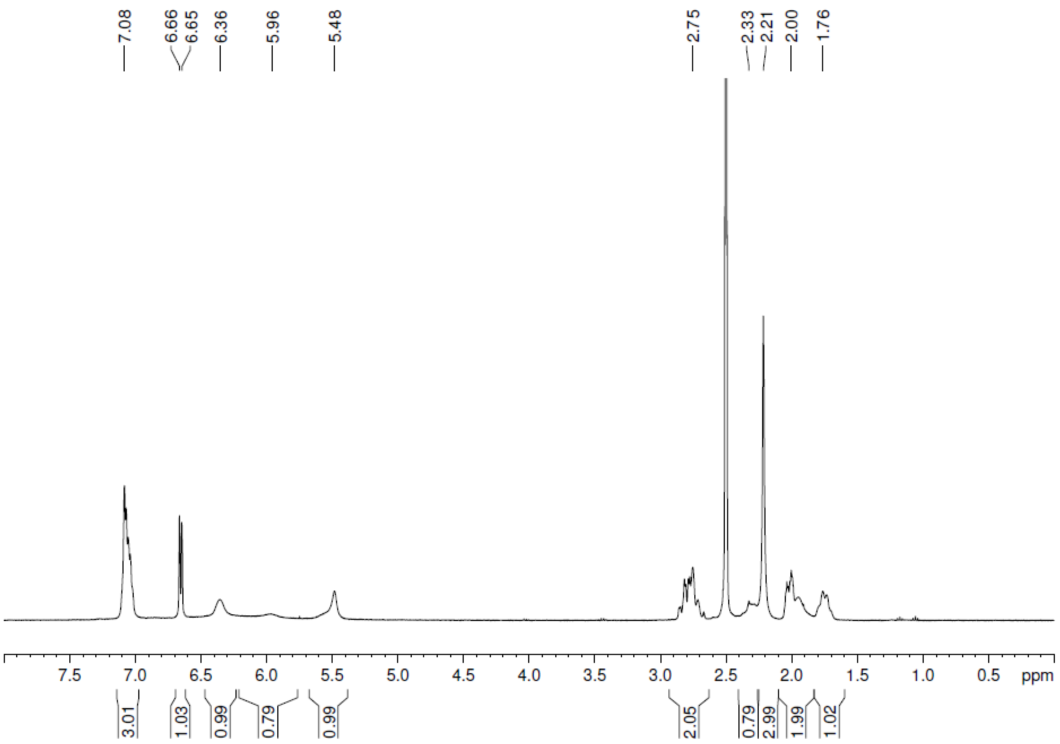

Fig. S13 ^{1}H NMR (400 MHz) spectrum of compound **3** in DMSO-d_6 at 295 K.

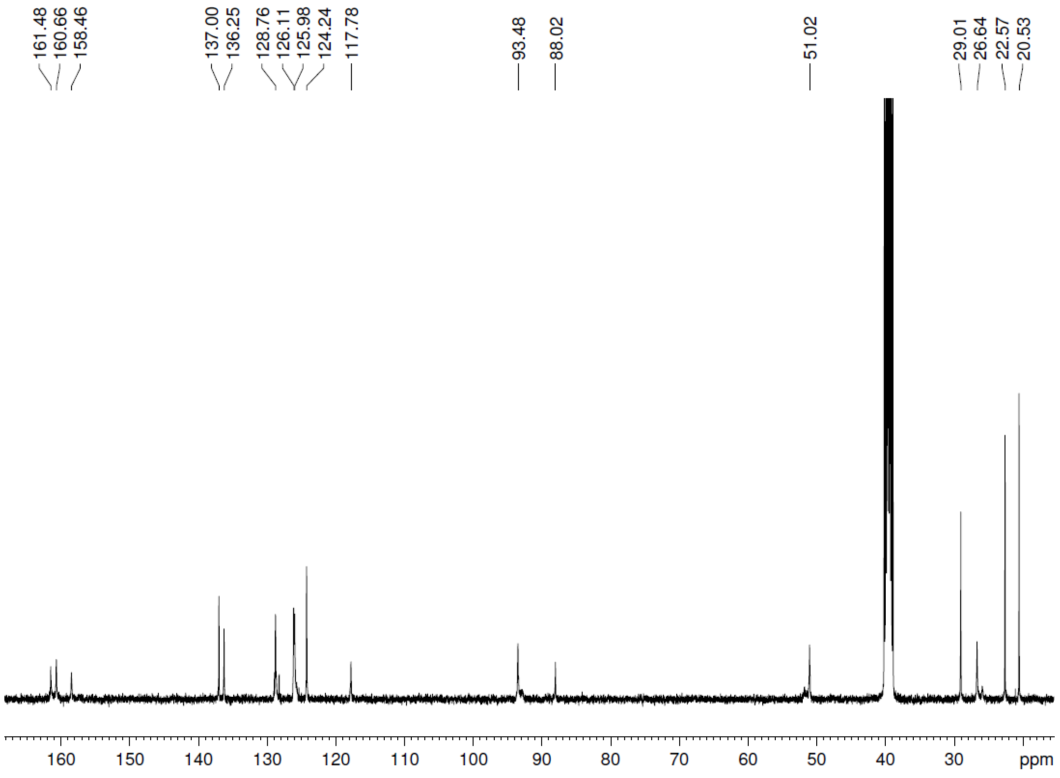

Fig. S14 ^{13}C NMR (101 MHz) spectrum of compound **3** in DMSO-d_6 at 295 K.

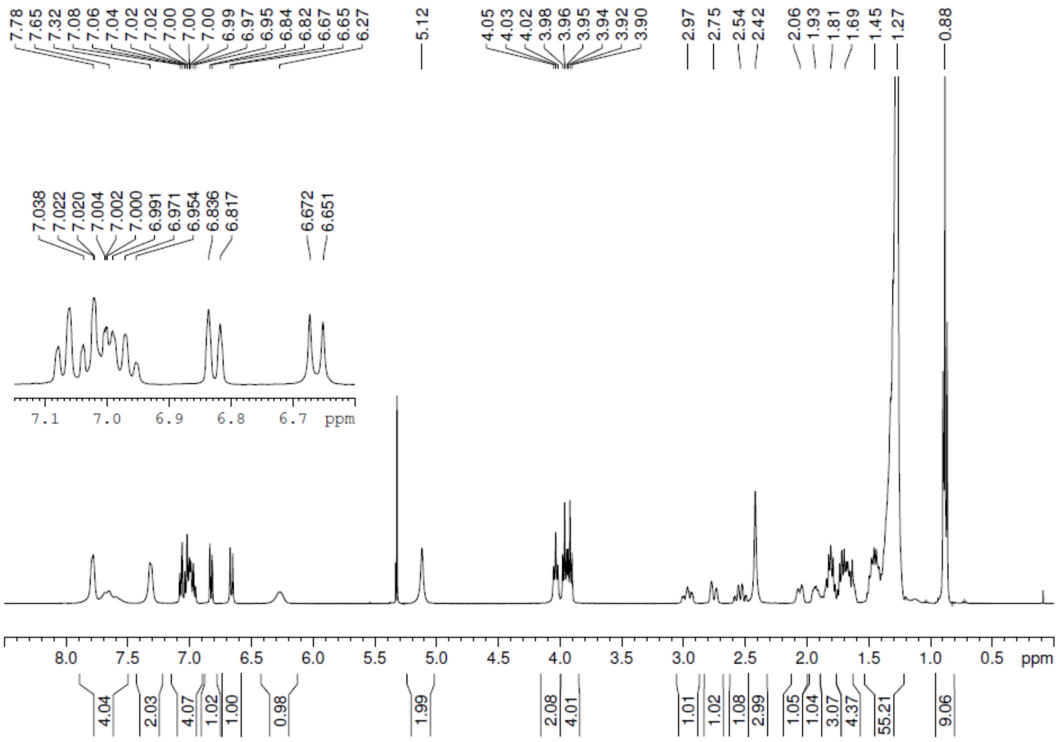

Fig. S15 ^{1}H NMR (400 MHz) spectrum of merocyanine **1** in CD_2Cl_2 at 295 K.

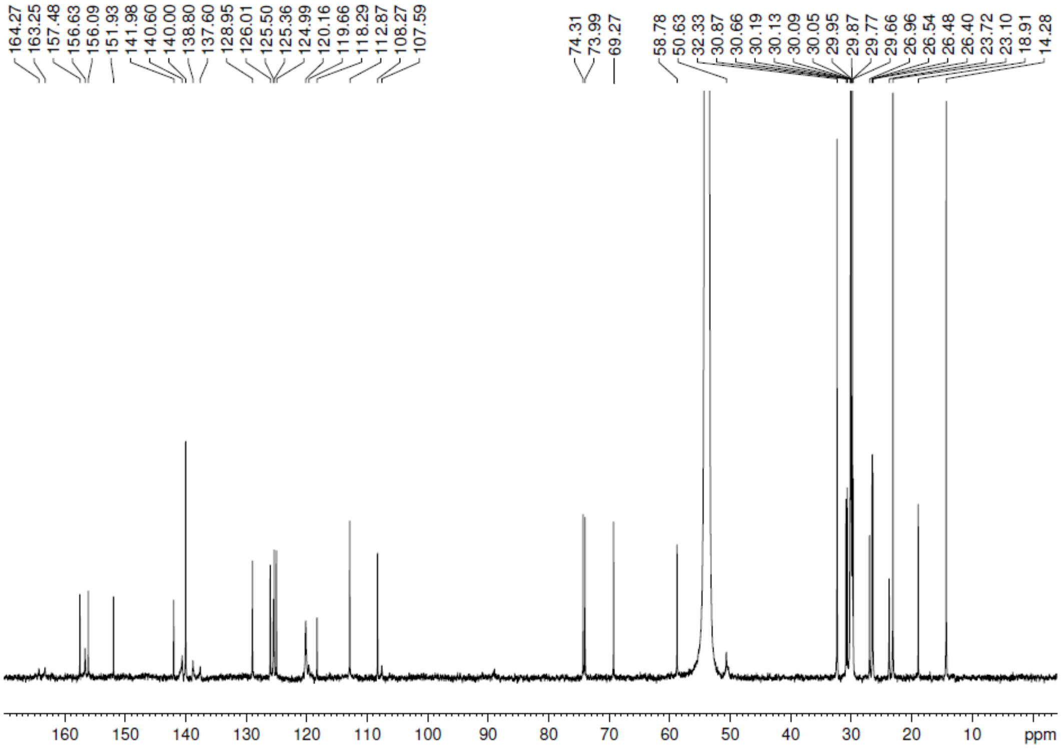

Fig. S16 ^{13}C NMR (151 MHz) spectrum of merocyanine **1** in CD_2Cl_2 at 295 K.

6.2 Proton Assignment and Structure Elucidation

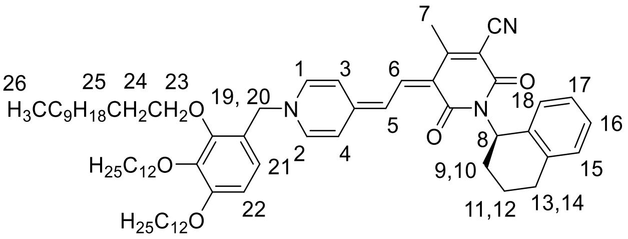

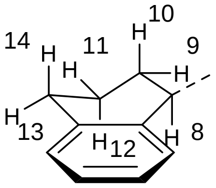

Table S2 Assignment of the ^{1}H and ^{13}C NMR signals of the monomer (M, CD_2Cl_2, 295 K) and dimer (D, MCH-d_{14}, 348 K) of merocyanine **1**.

Position	**M in CD_2Cl_2 (298 K)** δ_H /ppm (*J*/Hz)	δ_C /ppm	**D in MCH-d_{14} (348 K)** δ_H /ppm (*J* /Hz)	δ_C /ppm
1,2	7.78 (br, 2H)	140.00	7.30 (br, 2H)	140.8
3,4	7.32 (br, 2H)	120.16	6.87 (d, 6.4, 2H)	118.6
5	7.6 (br, 1H)	112.87	7.71 (d, 14.5, 1H)	113.2
6	7.7 (br, 1H)	140.60	7.38 (d, 14.5, 1H)	140.4
7	2.42 (s, 3H)	18.91	2.08 (s, 3H)	18.2
8	6.27 (br, 1H)	50.63	6.39 (t, 8.3, 1H)	49.9
9<br>10	1.93 (br, 1H)<br>2.54 (m, 1H)	26.96	1.91 (m, 1H)<br>2.60 (m, 1H)	27.6
11<br>12	2.06 (m, 1H)<br>1.81 (m, 1H)	23.72	2.05 (m, 1H)<br>1.79 (m, 1H)	24.6
13<br>14	2.75 (m, 1H)<br>2.97 (m, 1H)	30.18	2.67 (m, 1H)<br>3.11 (m, 1H)	30.8
15	7.07 (d, 7.4, 1H)	128.95	6.95 (m, 1H)	128.7
16	7.02 (t, 7.4, 1H)	125.50	6.89 (m, 1H)	125.7
17	6.97 (t, 7.4, 1H)	126.01	6.89 (m, 1H)	125.7
18	6.83 (d, 7.3, 1H)	124.99	6.93 (m, 1H)	126.2
19<br>20	5.12 (s, 2H)	58.78	5.16 (d, 14.0, 1H)<br>5.03 (d, 14.0, 1H)	58.9
21	6.95 (d, 8.5, 1H)	125.36	7.00 (d, 8.5, 1H)	126.88
22	6.66 (d, 8.5, 1H)	108.27	6.54 (d, 8.5,1H)	109.21
23	4.03 (t, 6.6, 2H)<br>3.96 (t, 6.4, 2H)<br>3.92 (t, 6.5, 2H)	74.31<br>69.27<br>73.99	4.00 – 3.89 (m, 6H)	74.2<br>69.6<br>73.9
24	1.81 (m, 2H)<br>1.70 (m, 4H)	29.66<br>30.66, 30.86	solvent overlap	
25	1.29 (m, 54)	23 – 32<br>(several peaks)	solvent overlap	
26	0.88 (t, 6.9, 9H)	14.28	solvent overlap	

6.2.1 Monomer – COSY and ROESY NMR

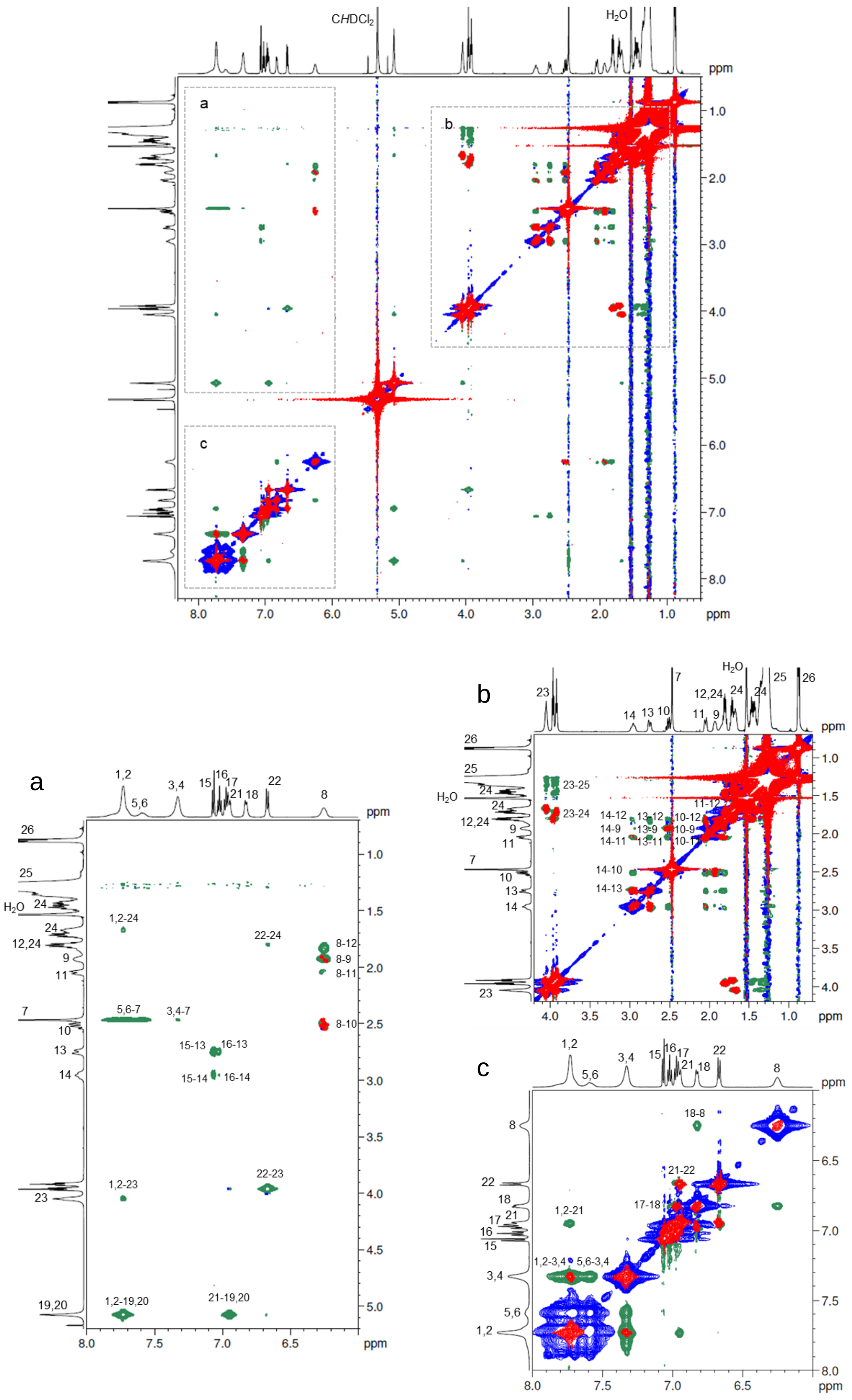

Fig. S17 ^{1}H ^{1}H ROESY NMR (600 MHz) spectrum of the monomer of merocyanine **1** ($c_0 = 1.4 \times 10^{-3}$ M, blue = positive/green = negative) in CD_2Cl_2 at 295 K overlaid with the ^{1}H ^{1}H COSY NMR (400 MHz) spectrum of **1** ($c_0 = 9.7 \times 10^{-4}$ M, red) in CD_2Cl_2 at 295 K. The sections a-c) are enlarged and the cross signals assigned to the respective protons as denoted in section 6.2.

6.2.2 Dimer – COSY and NOESY NMR

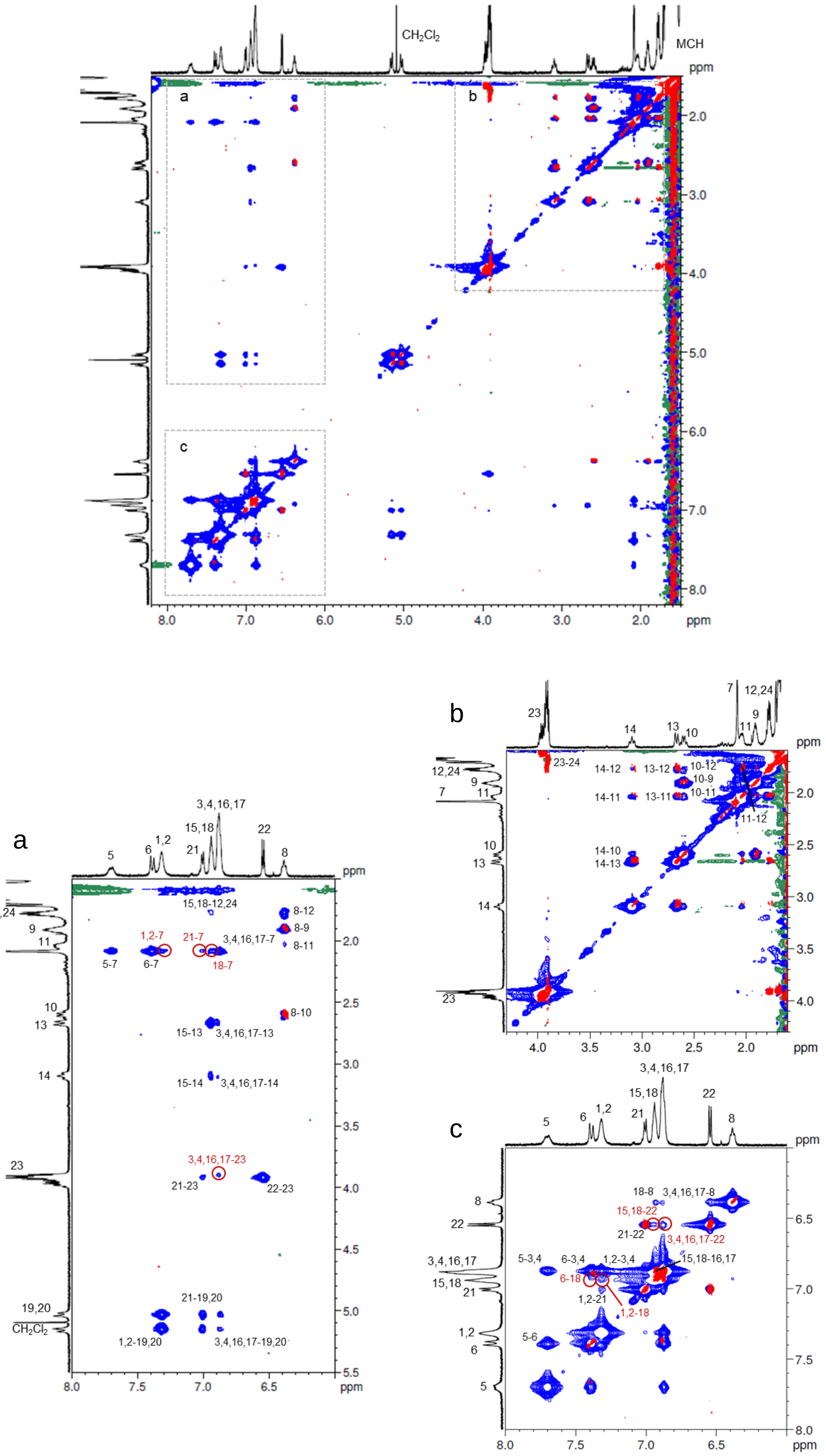

Fig. S18 ^{1}H ^{1}H NOESY NMR (600 MHz) spectrum of the dimer of merocyanine **1** ($c_0 = 2.7 \times 10^{-4}$ M, blue = positive/green = negative) in MCH-d_{14} at 348 K overlaid with the ^{1}H ^{1}H COSY NMR (600 MHz) spectrum of the dimer of **1** ($c_0 = 5.1 \times 10^{-4}$ M, red) in MCH-d_{14} at 348 K. The sections a-c) are enlarged and the cross signals assigned to the respective protons as denoted in section 6.2. Signals, that can not be explained by *intra*molecular proximities and therefore indicate *inter*molecular proximities between the two chromophores within the dimer are circled and labeled in red.

6.2.3 Monomer – HSQC NMR

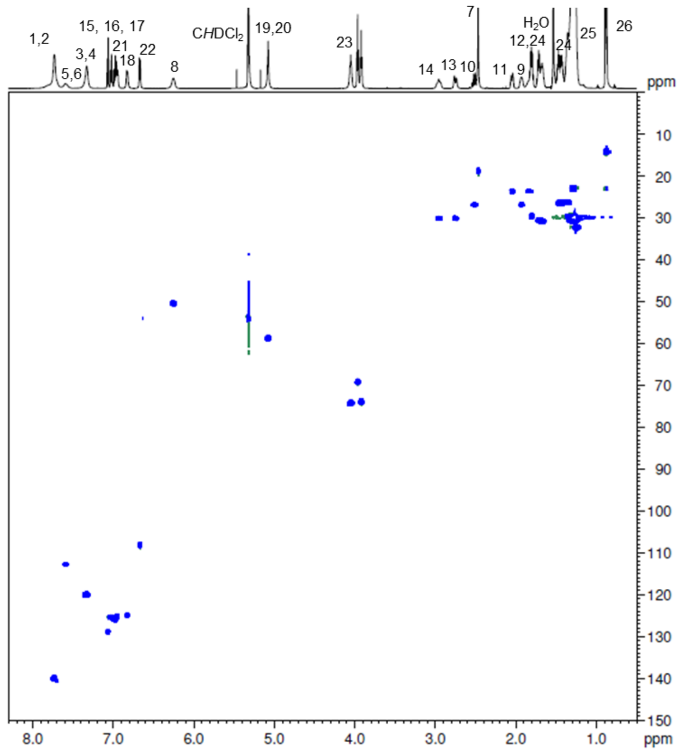

Fig. S19 ^{1}H ^{13}C HSQC NMR (600 MHz) spectrum of merocyanine **1** (c_0 = 1.4 × 10^{-3} M) in CD_2Cl_2 at 295 K.

6.2.4 Dimer – HSQC NMR

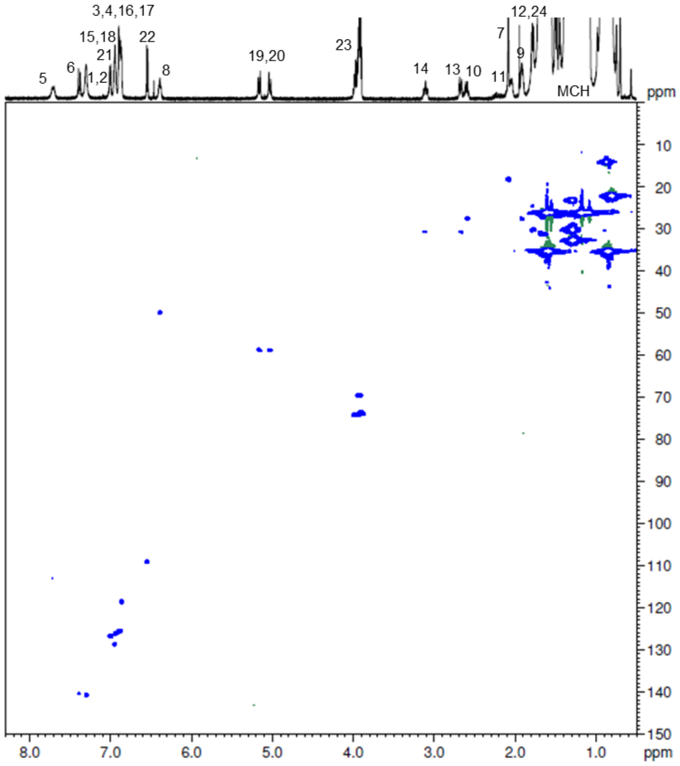

Fig. S20 ^{1}H ^{13}C HSQC NMR (600 MHz) spectrum of the dimer of merocyanine **1** (c_0 = 2.3 × 10^{-4} M) in MCH-d_{14} at 348 K.

6.2.5 Higher Aggregate – HSQC NMR

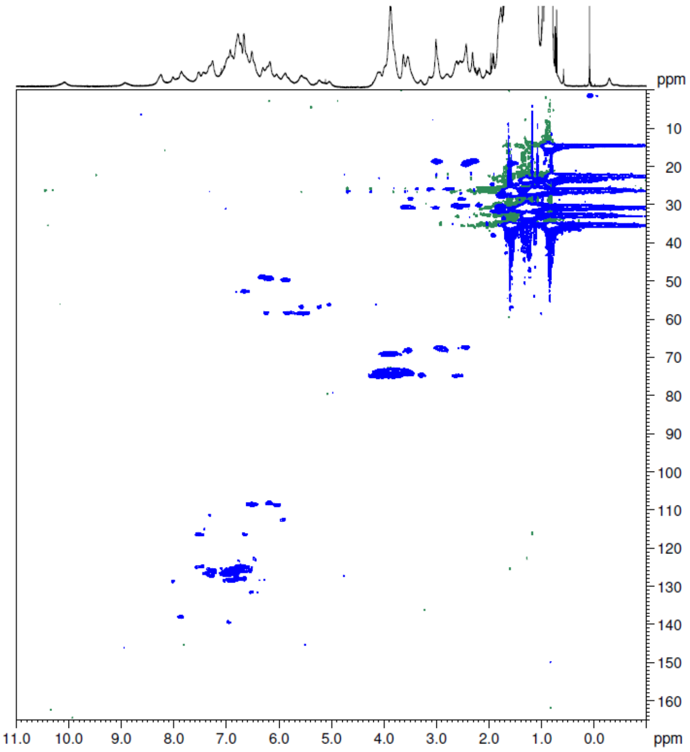

Fig. S21 ^{1}H ^{13}C HSQC NMR (600 MHz) spectrum of the higher aggregate of merocyanine **1** ($c_0 = 2.1 \times 10^{-3}$ M) in MCH-d_{14} at 295 K.

5.2 DOSY-Experiments

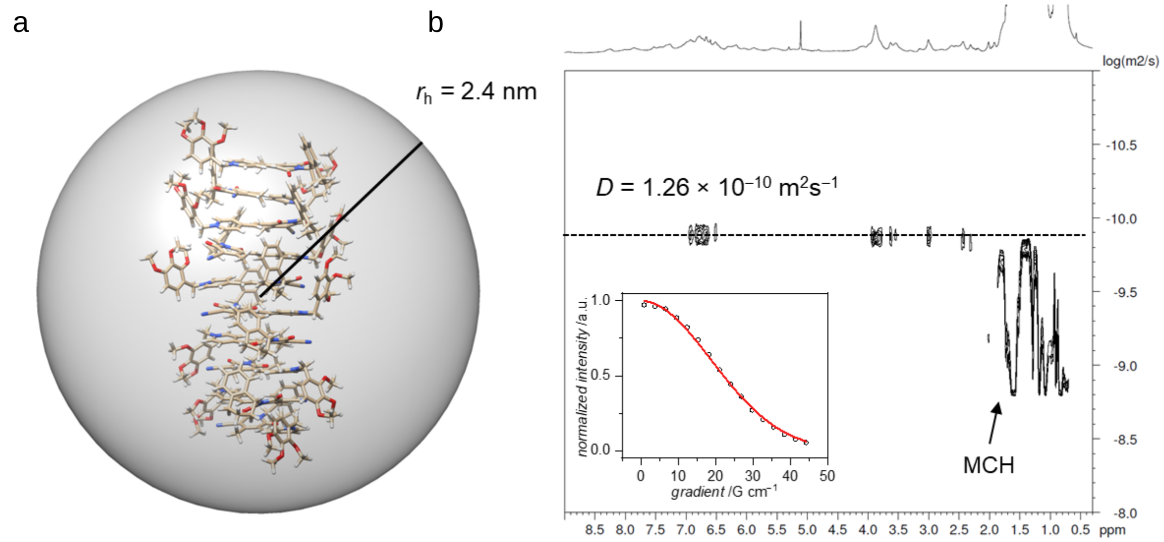

Fig. S22 Illustration of the geometry-optimized decamer stack of merocyanine **1** (PM7, dodecyl chains replaced by methyl groups) as well as the hydrodynamic radius (transparent grey sphere) as obtained from the Stokes-Einstein equation deduced from b) the ^{1}H DOSY NMR (600 MHz, 295 K) spectrum of **1** in MCH-d_{14} (c_0 = 1.0 × 10^{-3} M). Inset shows a representative attenuation curve for the integral from 6.86 – 6.62 ppm with the respective fit.

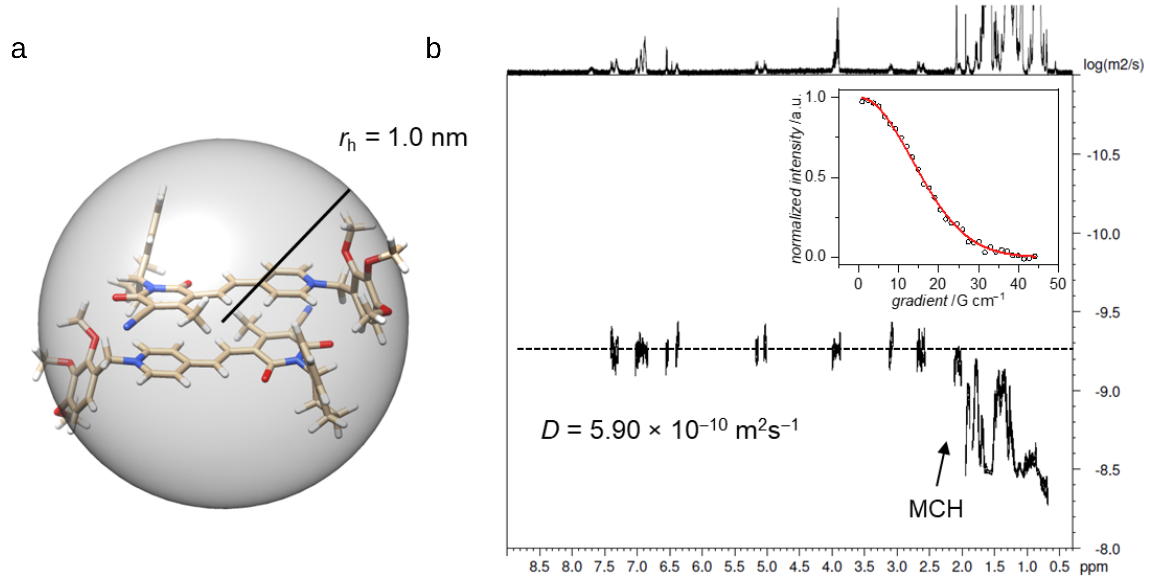

Fig. S23 Illustration of the geometry-optimized dimer structure of merocyanine **1** (B97D3/def2-SVP) as well as the hydrodynamic radius (transparent grey sphere) as obtained from the Stokes-Einstein equation deduced from b) the ^{1}H DOSY NMR (600 MHz, 348 K) spectrum of **1** in MCH-d_{14} (c_0 = 2.3 × 10^{-4} M). Inset shows a representative attenuation curve for the integral from 7.07 – 6.80 ppm with the respective fit.

7 CD Spectroscopy

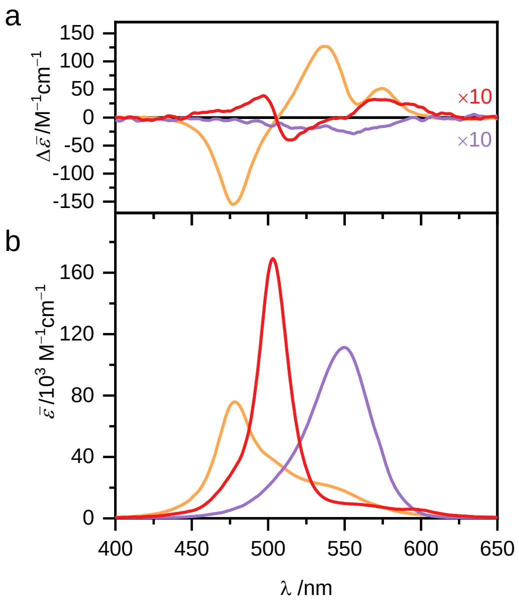

Fig. S24 a) CD and b) UV/Vis absorption spectra of the monomer ($c_0 = 7.2 \times 10^{-6}$ M, violet line) of merocyanine **1** in dichloromethane, as well as the dimer ($c_0 = 6.5 \times 10^{-6}$ M, red line) in MCH and the higher aggregate ($c_0 = 1.0 \times 10^{-3}$ M, orange line) in MCH at 298 K. The monomer and dimer CD spectra are depicted with ten-fold increased intensity.

8 Fluorescence Spectroscopy

8.1 Lifetime Measurements

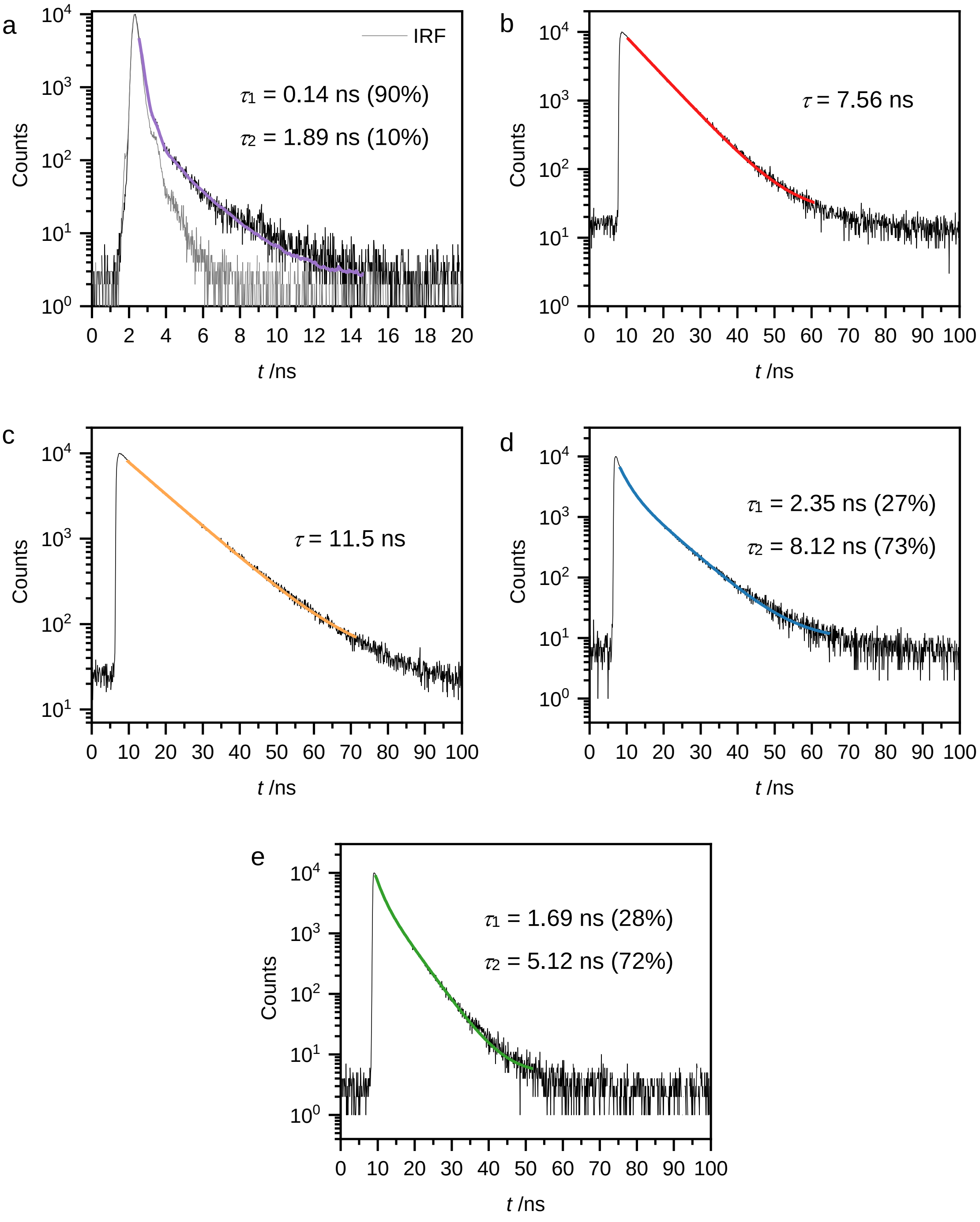

Fig. S25 Fluorescence lifetime measurements (λ_{ex} = 505.8 nm, 295 K) of merocyanine **1** samples with their respective mono-/bi-exponential fits: a) Monomer in CH_2Cl_2 ($c_0 = 3.9 \times 10^{-7}$ M) with instrument response function (IRF, grey). b) Dimer in MCH ($c_0 = 4.8 \times 10^{-6}$ M). c) Higher aggregate in MCH ($c_0 = 1.0 \times 10^{-3}$ M). d) Freeze-dried solid from cyclohexane solution. e) Solid from CH_2Cl_2 solution.

8.2 Relative Determination of the Higher Aggregate Fluorescence Quantum Yield

As the fluorescence quantum yield of the higher aggregate of merocyanine **1** could not be determined directly, it was estimated relative to the quantum yield of the dimer. For this, the spectral data of a dilution experiment were evaluated, which display the slow transition from higher aggregate to dimer after instant dilution from $c_0 = 1.0 \times 10^{-3}$ M to $c_0 = 1.0 \times 10^{-5}$ M over time (Fig. S26).

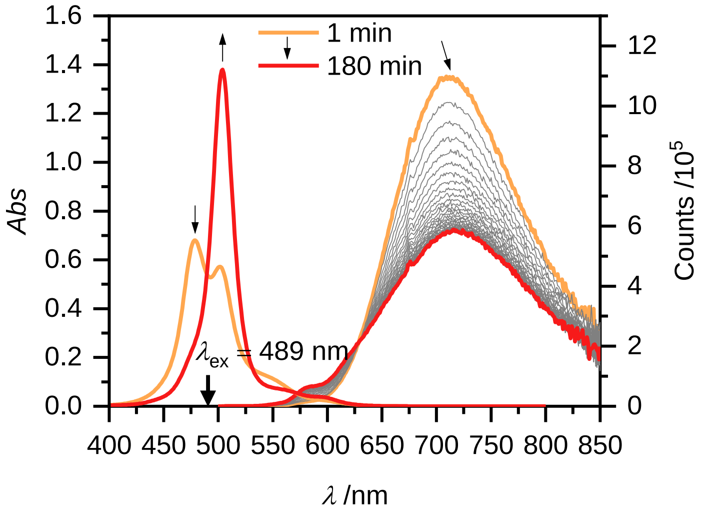

Fig. S26 Time-dependent UV/Vis absorption and fluorescence (λ_{ex} = 489 nm) spectra of merocyanine **1** in MCH at room temperature, 1 min (orange line) to 180 min (red line) after dilution from $c_0 = 1.0 \times 10^{-3}$ M to $c_0 = 1.0 \times 10^{-5}$ M.

Since the excitation wavelength was set at the isosbestic point at 489 nm, the sample always absorbs a constant amount of photons and the intensity of the emission can be directly correlated to the varying higher aggregate and dimer ratio. The fluorescence quantum yield Φ_{Fl} is proportional to the integral A of the emission spectrum (equation (S18)). A system of linear equations can be formulated as equations (S19) and (S20), where A_1 and A_2 are the areas under the emission band at two different times, which were chosen as 1 min (27% of dimer and 73% of higher aggregate) and 180 min (97% dimer and 3% higher aggregate) after dilution, respectively. x_H and x_D are the fraction of molecules present as higher aggregate (H) or dimer (D) at the observed time, according to multiple linear regression analysis of the corresponding absorption spectrum.

$$\Phi_{\mathrm{Fl}} \propto A . \tag{S18}$$

$$A_1 = x_{\mathrm{H1}} \cdot a_{\mathrm{H}} + x_{\mathrm{D1}} \cdot a_{\mathrm{D}} . \tag{S19}$$

$$A_2 = x_{\mathrm{H2}} \cdot a_{\mathrm{H}} + x_{\mathrm{D2}} \cdot a_{\mathrm{D}} . \tag{S20}$$

Accordingly, a_{H} and a_{D} are the integrals of the theoretical emission bands of 100 % higher aggregate or 100% dimer, respectively, which are proportional to the respective quantum yields of the species. Since the quantum yield of the dimer is known to be $\Phi_{\mathrm{Fl}}(D) = 2.3\%$, which is proportional to a_{D}, the quantum yield of the higher aggregate can be determined relative to this by solving the system of linear equations and comparing a_{D} and a_{H}. Φ_{Fl}(H) was determined by this procedure to be about 4.5%.

8.3 Solid State Fluorescence

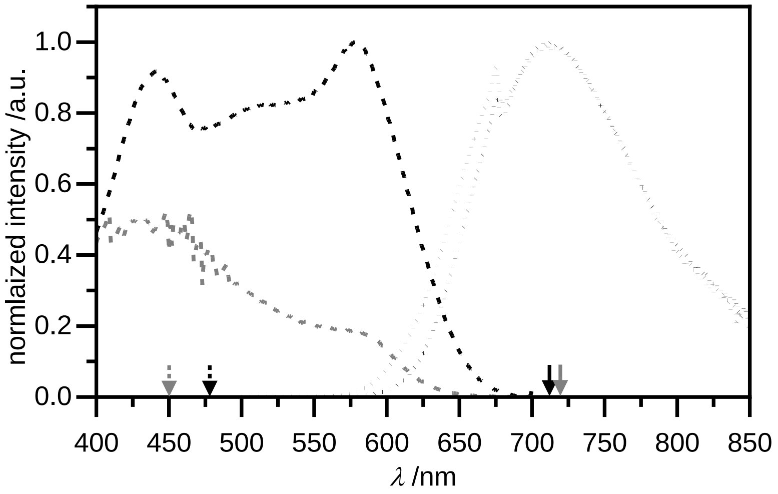

Fig. S27 Fluorescence (dotted line) and excitation (dashed line) spectra of solid samples of merocyanine **1**. Grey: evaporation of CH_2Cl_2 solution (λ_{ex} = 450 nm, λ_{em} = 720 nm). Black: Freeze-dried cyclohexane solution (λ_{ex} = 478 nm, λ_{em} = 712 nm). All recorded at room temperature with a front face setup.

9 Higher Aggregate Structure

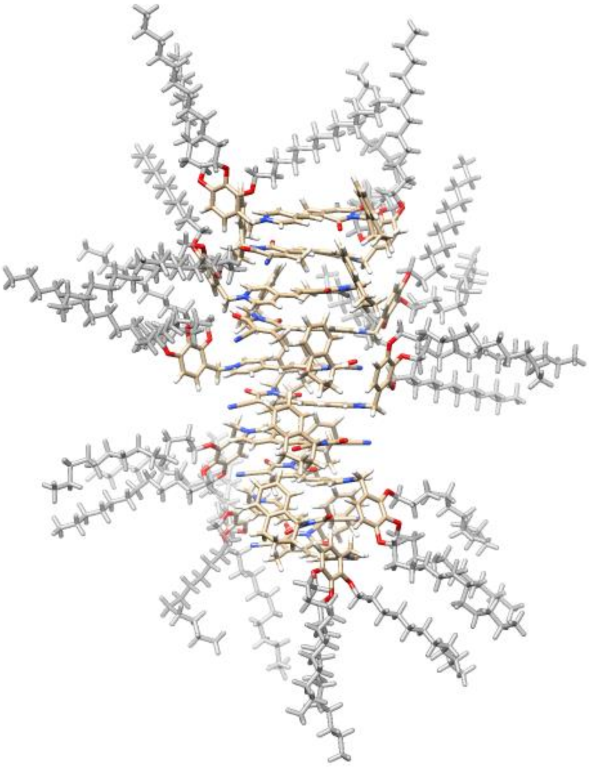

Fig. S28 Geometry-optimized *P*-helical decamer stack of merocyanine **1** (PM7). The dodecyl chains of the trialkoxyphenyl substituents were replaced by methyl groups for the calculation to reduce computational effort and added manually (in grey) to the optimized structure to illustrate the sterical demand of the solubilizing substituents.

10 Supporting References

S1. V. Percec, C. H. Ahn, T. K. Bera, G. Ungar and D. J. Yeardley, *Chem. Eur. J.*, 1999, **5**, 1070-1083.

S2. *China Pat.*, CN104263797, 2015.

S3. C. K. Zeberg-Mikkelsen, M. Barrouhou, A. Baylaucq and C. Boned, *High Temp. High Press.*, 2002, **34**, 591-601.

S4. G. Seybold and G. Wagenblast, *Dyes Pigm.*, 1989, **11**, 303-317.

S5. S. Grimme, J. Antony, S. Ehrlich and H. Krieg, *J. Chem. Phys.*, 2010, **132**, 154104.

S6. F. Weigend and R. Ahlrichs, *Phys. Chem. Chem. Phys.*, 2005, **7**, 3297-3305.

S7. Gaussian 16, Revision A.03, M. J. Frisch, G. W. Trucks, H. B. Schlegel, G. E. Scuseria, M. A. Robb, J. R. Cheeseman, G. Scalmani, V. Barone, G. A. Petersson, H. Nakatsuji, X. Li, M. Caricato, A. V. Marenich, J. Bloino, B. G. Janesko, R. Gomperts, B. Mennucci, H. P. Hratchian, J. V. Ortiz, A. F. Izmaylov, J. L. Sonnenberg, D. Williams-Young, F. Ding, F. Lipparini, F. Egidi, J. Goings, B. Peng, A. Petrone, T. Henderson, D. Ranasinghe, V. G. Zakrzewski, J. Gao, N. Rega, G. Zheng, W. Liang, M. Hada, M. Ehara, K. Toyota, R. Fukuda, J. Hasegawa, M. Ishida, T. Nakajima, Y. Honda, O. Kitao, H. Nakai, T. Vreven, K. Throssell, J. A. Montgomery, Jr., J. E. Peralta, F. Ogliaro, M. J. Bearpark, J. J. Heyd, E. N. Brothers, K. N. Kudin, V. N. Staroverov, T. A. Keith, R. Kobayashi, J. Normand, K. Raghavachari, A. P. Rendell, J. C. Burant, S. S. Iyengar, J. Tomasi, M. Cossi, J. M. Millam, M. Klene, C. Adamo, R. Cammi, J. W. Ochterski, R. L. Martin, K. Morokuma, O. Farkas, J. B. Foresman, and D. J. Fox, Gaussian, Inc., Wallingford CT, 2016.

S8. S. Miertuš, E. Scrocco and J. Tomasi, *Chem. Phys.*, 1981, **55**, 117-129.

S9. S. Miertuš̃ and J. Tomasi, *Chem. Phys.*, 1982, **65**, 239-245.

S10. J. L. Pascual-ahuir, E. Silla and I. Tuñon, *J. Comput. Chem.*, 1994, **15**, 1127-1138.

S11. J. J. Stewart, *J. Mol. Model.*, 2013, **19**, 1-32.

S12. MOPAC2016, Version: 18.346, J. J. P. Stewart, Stewart Computational Chemistry, Colorado Springs, CO, USA, 2016.

S13. J.-D. Chai and M. Head-Gordon, *J. Chem. Phys.*, 2008, **128**, 084106.

S14. R. Dennington, T. Keith, J. Millam, GaussView, Version 5 (Semichem Inc.), 2009.

S15. F. Würthner, S. Yao, T. Debaerdemaeker and R. Wortmann, *J. Am. Chem. Soc.*, 2002, **124**, 9431-9447.

S16. F. Fennel, S. Wolter, Z. Xie, P.-A. Plötz, O. Kühn, F. Würthner and S. Lochbrunner, *J. Am. Chem. Soc.*, 2013, **135**, 18722-18725.

S17. J. Gershberg, F. Fennel, T. H. Rehm, S. Lochbrunner and F. Würthner, *Chem. Sci.*, 2016, **7**, 1729-1737.

S18. E. Kirchner, D. Bialas, F. Fennel, M. Grüne and F. Würthner, *J. Am. Chem. Soc.*, 2019, **141**, 7428-7438.

S19. Z. Chen, A. Lohr, C. R. Saha-Möller and F. Würthner, *Chem. Soc. Rev.*, 2009, **38**, 564-584.
